# Supplementary material for: A hybrid CNN-SVM model for enhanced autism diagnosis
Source: PLoS One. 2024 May 14;19(5):e0302236. doi: 10.1371/journal.pone.0302236 (PMC11093301; doi:10.1371/journal.pone.0302236)
Supplement: Attachment — Submitted filename: PONE-D-23-32535_reviewer_commented.pdf [file pone.0302236.s001.pdf]

PLOS ONE

A hybrid CNN-SVM model for enhanced autism diagnosis

--Manuscript Draft--

**Manuscript Number:**	PONE-D-23-32535
**Article Type:**	Research Article
**Full Title:**	A hybrid CNN-SVM model for enhanced autism diagnosis
**Short Title:**	CNN-SVM model for autism diagnosis
**Corresponding Author:**	Linjie Qiu<br>Zhejiang University<br>Hangzhou, CHINA
**Keywords:**	autism; functional connectivity; Social Responsiveness Scale; convolutional neural networks; support vector machine; attention mechanisms
**Abstract:**	Autism is a representative disease of pervasive developmental disorder. Currently, most scholars' research primarily relies on neuroimaging techniques for auxiliary diagnosis and does not take into account of the distinctive features of autism's social impediments. This paper introduces a novel convolutional neural network-support vector machine model that integrates resting state functional magnetic resonance imaging data with the Social Responsiveness Scale metrics for the diagnostic assessment of autism. Utilizing convolutional neural networks and attention mechanisms, the model extracts features from both static and dynamic functional connectivity. The extracted features, combined with the Social Responsiveness Scale features, are then employed as novel inputs for the support vector machine to categorize autistic patients and typical controls. The proposed model identifies salient features within the static and dynamic functional links, offering a possible biological foundation for clinical diagnosis. By incorporating the behavioral assessments, the model achieves a remarkable classification accuracy of 94.30%, providing a more reliable support for auxiliary diagnosis.
**Order of Authors:**	Linjie Qiu
	Jian Zhai
**Additional Information:**	
**Question**	**Response**
**Financial Disclosure**<br><br>Enter a financial disclosure statement that describes the sources of funding for the work included in this submission. Review the submission guidelines for detailed requirements. View published research articles from *PLOS ONE* for specific examples.<br><br>This statement is required for submission and **will appear in the published article** if the submission is accepted. Please make sure it is accurate.	Yes

Powered by Editorial Manager® and ProduXion Manager® from Aries Systems Corporation

**Funded studies**<br>Enter a statement with the following details:<br>• Initials of the authors who received each award<br>• Grant numbers awarded to each author<br>• The full name of each funder<br>• URL of each funder website<br>• Did the sponsors or funders play any role in the study design, data collection and analysis, decision to publish, or preparation of the manuscript?<br><br>Did you receive funding for this work?	
Please add funding details.<br>as follow-up to "**Financial Disclosure**<br><br>Enter a financial disclosure statement that describes the sources of funding for the work included in this submission. Review the submission guidelines for detailed requirements. View published research articles from *PLOS ONE* for specific examples.<br><br>This statement is required for submission and **will appear in the published article** if the submission is accepted. Please make sure it is accurate.<br><br>**Funded studies**<br>Enter a statement with the following details:<br>• Initials of the authors who received each award<br>• Grant numbers awarded to each author<br>• The full name of each funder<br>• URL of each funder website<br>• Did the sponsors or funders play any role in the study design, data collection and analysis, decision to publish, or preparation of the manuscript?<br><br>Did you receive funding for this work?"	This work was supported by the National Natural Science Foundation of China under Grant numbers 11671354.
Please select the country of your main research funder (please select carefully as in some cases this is used in fee calculation).	CHINA - CN

as follow-up to "**Financial Disclosure**<br><br>Enter a financial disclosure statement that describes the sources of funding for the work included in this submission. Review the submission guidelines for detailed requirements. View published research articles from *PLOS ONE* for specific examples.<br><br>This statement is required for submission and **will appear in the published article** if the submission is accepted. Please make sure it is accurate.<br><br>**Funded studies**<br>Enter a statement with the following details:<br>• Initials of the authors who received each award<br>• Grant numbers awarded to each author<br>• The full name of each funder<br>• URL of each funder website<br>• Did the sponsors or funders play any role in the study design, data collection and analysis, decision to publish, or preparation of the manuscript?<br><br>Did you receive funding for this work?"	
**Competing Interests**<br><br>Use the instructions below to enter a competing interest statement for this submission. On behalf of all authors, disclose any competing interests that could be perceived to bias this work—acknowledging all financial support and any other relevant financial or non-financial competing interests.<br><br>This statement is required for submission and **will appear in the published article** if the submission is accepted. Please make sure it is accurate and that any funding sources listed in your Funding Information later in the submission form are also declared in your Financial Disclosure statement.	NO authors have competing interests

View published research articles from *PLOS ONE* for specific examples.<br><br>**NO authors have competing interests**<br><br>Enter: *The authors have declared that no competing interests exist.*<br><br>**Authors with competing interests**<br><br>Enter competing interest details beginning with this statement:<br><br>*I have read the journal's policy and the authors of this manuscript have the following competing interests: [insert competing interests here]*<br><br>* typeset	
**Ethics Statement**<br><br>Enter an ethics statement for this submission. This statement is required if the study involved:<br><br>• Human participants<br>• Human specimens or tissue<br>• Vertebrate animals or cephalopods<br>• Vertebrate embryos or tissues<br>• Field research<br><br>Write "N/A" if the submission does not require an ethics statement.<br><br>General guidance is provided below. Consult the submission guidelines for detailed instructions. **Make sure that all information entered here is included in the Methods section of the manuscript.**	N/A

Powered by Editorial Manager® and ProduXion Manager® from Aries Systems Corporation

**Format for specific study types**<br><br>**Human Subject Research (involving human participants and/or tissue)**<br>• Give the name of the institutional review board or ethics committee that approved the study<br>• Include the approval number and/or a statement indicating approval of this research<br>• Indicate the form of consent obtained (written/oral) or the reason that consent was not obtained (e.g. the data were analyzed anonymously)<br><br>**Animal Research (involving vertebrate animals, embryos or tissues)**<br>• Provide the name of the Institutional Animal Care and Use Committee (IACUC) or other relevant ethics board that reviewed the study protocol, and indicate whether they approved this research or granted a formal waiver of ethical approval<br>• Include an approval number if one was obtained<br>• If the study involved *non-human primates*, add *additional details* about animal welfare and steps taken to ameliorate suffering<br>• If anesthesia, euthanasia, or any kind of animal sacrifice is part of the study, include briefly which substances and/or methods were applied<br><br>**Field Research**<br><br>Include the following details if this study involves the collection of plant, animal, or other materials from a natural setting:<br>• Field permit number<br>• Name of the institution or relevant body that granted permission	
**Data Availability**<br><br>Authors are required to make all data underlying the findings described fully available, without restriction, and from the time of publication. PLOS allows rare exceptions to address legal and ethical concerns. See the PLOS Data Policy and FAQ for detailed information.	Yes - all data are fully available without restriction

A Data Availability Statement describing where the data can be found is required at submission. Your answers to this question constitute the Data Availability Statement and **will be published in the article**, if accepted.<br><br>**Important:** Stating 'data available on request from the author' is not sufficient. If your data are only available upon request, select 'No' for the first question and explain your exceptional situation in the text box.<br><br>Do the authors confirm that all data underlying the findings described in their manuscript are fully available without restriction?	
**Describe where the data may be found in full sentences. If you are copying our sample text, replace any instances of XXX with the appropriate details.**<br><br>• If the data are **held or will be held in a public repository**, include URLs, accession numbers or DOIs. If this information will only be available after acceptance, indicate this by ticking the box below. For example: *All XXX files are available from the XXX database (accession number(s) XXX, XXX.).*<br>• If the data are all contained **within the manuscript and/or Supporting Information files**, enter the following: *All relevant data are within the manuscript and its Supporting Information files.*<br>• If neither of these applies but you are able to provide **details of access elsewhere**, with or without limitations, please do so. For example:<br><br>*Data cannot be shared publicly because of [XXX]. Data are available from the XXX Institutional Data Access / Ethics Committee (contact via XXX) for researchers who meet the criteria for access to confidential data.*<br><br>*The data underlying the results presented in the study are available from (include the name of the third party*	The datasets we used are common public datasets from URL: http://fcon_1000.projects.nitrc.org/indi/abide/

and contact information or URL).<br>• This text is appropriate if the data are owned by a third party and authors do not have permission to share the data.<br><br>* typeset	
Additional data availability information:	

Click here to access/download;Manuscript;plos_latex_manuscript.pdf

A hybrid CNN-SVM model for enhanced autism diagnosis

Linjie Qiu[1*☯], Jian Zhai[1☯]

1 School of Mathematical Sciences, Zhejiang University, Hangzhou, Zhejiang, China

☯These authors contributed equally to this work.
* 11935034@zju.edu.cn

Abstract

Autism is a representative disease of pervasive developmental disorder. Currently, most scholars' research primarily relies on neuroimaging techniques for auxiliary diagnosis and does not take into account of the distinctive features of autism's social impediments. This paper introduces a novel convolutional neural network-support vector machine model that integrates resting state functional magnetic resonance imaging data with the Social Responsiveness Scale metrics for the diagnostic assessment of autism. Utilizing convolutional neural networks and attention mechanisms, the model extracts features from both static and dynamic functional connectivity. The extracted features, combined with the Social Responsiveness Scale features, are then employed as novel inputs for the support vector machine to categorize autistic patients and typical controls. The proposed model identifies salient features within the static and dynamic functional links, offering a possible biological foundation for clinical diagnosis. By incorporating the behavioral assessments, the model achieves a remarkable classification accuracy of 94.30%, providing a more reliable support for auxiliary diagnosis.

Introduction

Autism spectrum disorder (ASD) is a neurodevelopmental disease marked by language impairment, social impairment, and stereotyped behavior. According to statistical data, approximately one in every 100 children is diagnosed with autism [1]. Despite its characteristics often being identifiable in early childhood, definitive diagnosis typically requires an extended period of time. Given that individuals with ASD generally need specialized medical care to mitigate the risk of co-occurring mental health conditions, timely and accurate diagnosis is imperative.

The intricate physiological activities within the human body orchestrated through the coordinated interplay among various brain regions, and the relationship between brain regions evolves over time. Leveraging these connections holds promise for diagnosis of autism and finding areas that causes brain disorders. Resting-state functional magnetic resonance imaging (rs-fMRI), with its advantages of high spatio-temporal resolution and absence of a requirement for elaborate tasks [2, 3], has been extensively utilized for investigating a wide array of psychiatric conditions. Constructing functional connectivity (FC) based rs-fMRI serves as an efficient method for characterizing relationships among brain areas. Early scholarly articles often focused solely on static functional connectivity, which presupposes that brain interrelations remain constant throughout the whole scanning process. However, recent literature reveals that neural connections fluctuate during the scan, thus giving rise to the broader application of dynamic functional connectivity in brain activity research [4–6]. To

furnish a more comprehensive evaluation of the brain activities, this paper employs both static and dynamic functional connectivity to analyze differences between the subjects with ASD and typical controls (TCs), aiming to identify areas causing ASD. Nevertheless, only the analysis of brain activity may prove insufficient for the accurate diagnosis of ASD, necessitating the incorporation of additional information.

Current psychiatric screening or diagnostic procedures predominantly rely on behavioral observations rooted in symptomatology. For example, the Social Responsiveness Scale–Second Edition (SRS-2) serves as an early screening tool for ASD [7], quantifying social functionality in five aspects: awareness, cognition, communication, motivation, and mannerisms. Substantial evidence supports its effectiveness and sensitivity in identifying autism symptoms in school-age children. However, the questionnaire mainly focuses on social communication, and only a few items involve stereotyped behavior. Researchers are still gathering more data on the stability and effectiveness of the SRS-2 [8–10]. While other diagnostic tools such as the Autism Diagnostic Observation Schedule, the Childhood Autism Rating Scale, and the Autism Diagnostic Interview-Revised are commonly employed in clinical settings, these methods are time-consuming. To promote more accurate diagnosis, it is not enough to only use the scale results to assist diagnosis. Consequently, we have integrated analyses of functional connectivity to better assist diagnosis.

Machine learning is widely used for diagnostic assistance, including traditional machine learning, such as support vector machines (SVM), random forests (RF), and deep learning models, such as convolutional neural networks (CNN). While traditional machine learning algorithms demonstrate commendable performance in classification tasks, their accuracy still needs to be improved, and deep features cannot be extracted. On the other hand, deep learning models can acquire deep features and offer improvements in classification accuracy, but these models still transfer the processing of general images when processing functional connectivity, without adapting to the unique characteristics of functional connectivity.

To harness the full potential of early screening questionnaires and brain functional connectivity for autism diagnosis, this paper introduces a hybrid CNN-SVM model. This model employs a CNN architecture to extract deep features from both static and dynamic functional connectivity. In the learning process, the feature extraction based on the frequency band and the convolution kernel based on the functional connectivity matrix are used, and the attention mechanism is introduced to weight the learned features. Finally, the learned features combined with the features from the SRS are sent to SVM for classification. After studying 379 subjects with ASD and 442 typical controls, our model achieves good classification accuracy and provides the most discriminating brain regions and scanning bands.

Materials and methods

The whole process of this experiment is illustrated in Figure 1. More details of Data preprocessing, staticCNN and dynamicCNN are shown in the following sections.

Fig 1. The entire process of this experiment. Details of the staticCNN and dynamicCNN part are illustrated in Figure 2 and Figure 3 separately.

0.1 Data preprocessing

Firstly, we select resting-state fMRI data that includes SRS metrics from the publicly available Autism Brain Imaging Data Exchange (ABIDE) dataset [11]. The distribution

and age of the subjects selected are shown in the Table 1. Upon acquiring the data from the ABIDE dataset, we perform the following preprocessing steps:

1. Extract the evaluation scores of the five aspects of the SRS, convert them into a one-dimensional vector and subsequently sent it to the final SVM for classification, as depicted in the SRS feature in Figure 1.

2. Remove the first ten time points of each subject to mitigate errors caused by the instability of the gradient magnetic field at the beginning of the scan. Correct slice scan time to ensure that the resampled data can be seen as scanned at the same time point. Carry out head motion correction to mitigate the impact of head movements to some extent. Perform nuisance regression to eliminate the influence of extraneous factors.

3. Register the structural image data and then map it to the standard space-MNI space. This aligns the images of each subject to ensure that the anatomical structures of different subjects correspond to the same voxels. Perform spatial smoothing to enhance registration effects.

4. Employ band–pass filtering (0.01–0.08 Hz). Most nuclear magnetic resonance signals are low–frequency signals. Given that magnetic resonance signals are primarily low–frequency signals, this frequency range is chosen to minimize the impact of physiological noise from cardiac (~ 0.15) and respiratory ($\sim$0.3 Hz) activities [12].

5. Utilize the Automated Anatomical Labeling (AAL) atlas, which partitions the brain into 116 regions. These regions serve as our regions of interest (ROIs), and the corresponding time series are extracted.

This study uses SPM12 and DPARSF [13] to preprocess fMRI data as mentioned above, and the extracted time series is used for subsequent processing.

Table 1. Distribution of the data from rs-fMRI ABIDE database used in this study.

LEUVEN	29	35	64
BNI	28	29	57
ETH	11	22	33
GU	51	55	106
IU	15	11	26
KKI	52	148	200
NYU	75	28	103
OHSU	33	46	79
SDSU	32	25	57
TCD	21	21	42
UCD	18	14	32
USM	14	8	22
total	379	442	821

0.2 static functional connectivity

The construction of a static functional connectivity matrix is predicated on the Pearson correlation coefficients between various regions of interest in the brain, as shown in Eq 1.

$$R(X,Y) = \frac{\sum(x_i - \overline{x}_i)(y_i - \overline{y}_i)}{\sqrt{\sum(x_i - \overline{x}_i)^2}\sqrt{\sum(y_i - \overline{y}_i)^2}}, \tag{1}$$

where X and Y are two distinct brain regions, while x_i and y_i denote the corresponding time series. $\overline{x}_i$ and $\overline{y}_i$ are mean values of x_i and y_i separately. The underlying assumption of static functional connectivity is that the interconnections between different brain areas remain invariant throughout the entire scanning procedure, and the Pearson correlation coefficient characterizes this relationship [5]. The resulting static

functional connectivity matrix has dimensions of 116×116, as illustrated in the input part of Figure 2.

0.3 dynamic functional connectivity

Throughout the scanning process, the functional connectivity between different brain regions exhibits temporal fluctuations, indicating that a representation solely based on static functional connectivity would be overly simplistic. This has led to the introduction of dynamic functional connectivity, which also serves to enrich the data set. Sliding-window analysis is a method often considered for dynamic functional connectivity. It describes changes in the brain's connection patterns by using a fixed window size and moving it at a specific stride. However, there is no consensus on the optimal size for the fixed temporal window. Additionally, variations have been observed in connectivity matrices at different frequencies [14, 15]. These issues cannot be solved by sliding-window method. We therefore perform a time-frequency analysis of the signal. We choose to use wavelet analysis to characterize dynamic functional connections, which effectively extracts information from signals through operations such as scaling and translation, thus enabling a multi-scale, fine-grained examination.

Initially, we employ continuous wavelet transforms to process the time series for each brain area, as delineated by the Eq 2.

$$W(s,\tau) = \frac{1}{\sqrt{s}} \int_{-\infty}^{\infty} x(t)\phi^{*}(\frac{t-\tau}{s})dt, \tag{2}$$

where s is the wavelet scale, τ denotes the translation value, $x(t)$ is the signal, ϕ represents a mother wavelet and $*$ denotes the complex conjugate [16]. Scale s controls the expansion and contraction of the wavelet function, which is inversely proportional to frequency. The translation τ controls the translation of the wavelet function, which corresponds to time points. When we traverse the required scales and translations, we can obtain the needed spectrogram from the unstable signal for subsequent analysis. We employ the Morlet wavelet as the mother wavelet, which offers an optimal ratio between frequency bands and wavelet scales, facilitating the understanding of data within specific frequency ranges [17]. In our analysis, we partition the frequency range of 0.01-0.08 Hz into 40 segments, and the translation τ corresponds to the sampled time points.

After obtaining the time-frequency spectra for each region of interest, we proceed to compute their dynamic functional connectivity. Here, in order to describe the time-frequency spatial relationship between two signals, we initially introduce the concept of cross-wavelet power [16], which identifies regions of the common high power between the two signals, as shown in Eq 3.

$$CW_{xy}(s,\tau) = W_x(s,\tau)W_y^{*}(s,\tau), \tag{3}$$

where W_x and W_y are the continuous wavelet of x and y separately. To further describe the coherence of these two cross wavelets in time-frequency space as the functional connectivity between two brain areas, we introduce the concept of wavelet coherence [16], calculated as Eq 4.

$$R_{xy} = \frac{|S(CW_{xy}(s,\tau))|^2}{S(|W_x(s,\tau)|^2) \cdot S(|W_y(s,\tau)|^2)}, \tag{4}$$

where $S(\cdot)$ denotes a smoothing operator related to scale and time, for which we employ the most commonly used moving average technique.

Finally, after processing different times and scales, we convert the signals of any two regions of interest into a matrix $WC \in R^{m \times n}$, here m corresponds to the number of

chosen scales, specifically $m = 40$,and n corresponds to the number of time points. In this way, for the same individual, we can get 6612 completely different matrices. Obviously, the amount of data is too large. At the same time, the data obtained by different institutions have different time points n, and so they cannot be easily passed to the same neural network for learning. To this end, we consider employing Principal Component Analysis (PCA) on the matrices [18]. In order to facilitate the unification of data from different institutions, we opt to perform dimensionality reduction on the temporal dimension, as detailed in the following procedures:

1. Calculate wavelet coherence for two regions of interest to obtain a matrix $WC = [wc_1, ..., wc_n]$

2. For the matrix WC, remove the average value to get $\hat{WC} = [w_1, ..., w_n]$, where $w_i = wc_i - \sum_{j=1}^{n} wc_i/n, i = 1, 2, ..., n.$

3. Construct matrix $\frac{1}{n}\hat{WC} \cdot \hat{WC}^T$. Use the eigenvalue decomposition method to calculate its eigenvalues and the corresponding unit eigenvectors. Suppose there are l eigenvectors and unit eigenvalues, and $\lambda_1, \lambda_2, ..., \lambda_l$ are arranged from large to small. Then $\frac{1}{n}\hat{WC}\hat{WC}^T X_i = \lambda_i X_i, i = 1, 2, ..., l.$

4. In order to retain 99% of the information, it is necessary to find the smallest k that satisfies $\frac{\sum_{i=1}^{k} \lambda_i}{\sum_{i=1}^{l} \lambda_i} \geq 0.99$. These k unit eigenvectors can form an eigenvector matrix P.

5. Finally, the matrix is mapped to the space composed of the corresponding feature vectors, that is, $XP \in R^{m \times k}$.

After performing principal component analysis on all matrices $WC \in R^{m \times n}$, we find that $k = 1$ can satisfy the condition in step 4. Thus we finally obtain a matrix of $116 \times 116 \times 40$ for each subject as the dynamic functional connectivity.

0.4 neural network

As illustrated in Figure 1, the input to our model is based on data from SRS tables, static FC and dynamic FC. Here, we employ CNNs to extract features from static FC and dynamic FC. For ordinary pictures, feature extraction is proficiently achieved through the utilization of 3×3 or 5×5 convolution kernels [19, 20]. However, when it comes to an individual FC matrix, each row or column corresponding to the correlation between one brain area and another brain area. The aforementioned convolution kernel cannot summarize such information, and there is no reasonable explanation for the local features. Therefore, we believe that a more reasonable convolution kernel should be $1 \times n$ or $n \times 1$, where n corresponds to the number of regions of interest, that is, 116. The convolution kernel proposed here is more capable of extracting brain areas corresponding to functional connections that make greater contributions to classification.

For static FC, each subject corresponds to a $116 \times 116 \times 1$ tensor, as shown in Fig 2. We send it into the CNN. The first layer comprises 32 filters, and the corresponding convolution kernel is 1×116. After passing through the batch normalization (BN) layer and rectified linear unit (ReLu) layer, it is sent to the second convolution layer. The second layer comprises 64 filters, and the corresponding convolution kernel is 116×1. After passing through the BN layer and ReLu layer, it is sent to the third convolution layer. The second layer comprises 8 filters, and the corresponding convolution kernel is 1×1. Finally, it goes through the BN layer and ReLu layer. The third layer of convolutional layer is to prevent the extracted feature dimension from being too large and affecting subsequent classification. In addition to this step, we also use a dropout layer to prevent overfitting, where the dropout rate is set to 50%. All activation functions employed are of the LeakyReLU type ($\alpha = 0.01$).

The most discriminative deep features need to be distinguished through the attention mechanism [21], which follows the dropout layer. This attention network is bifurcated into two fully connected layers: the first boasting 8 neurons, followed by a ReLU layer, and the second containing 16 neurons, which is the same number of the input neurons. Then, it passes through the Sigmoid function to obtain the normalized weight of each input feature. These normalized weights are utilized for weighted summation to extract the requisite static functional connectivity features. To facilitate backpropagation for parameter tuning, an additional layer is added, the size of which corresponds to the number of classification categories, which is 2. The loss between the actual labels and the predicted outcomes is minimized via the adaptive moment estimation optimizer , with a learning rate set at 0.0001. The employed loss function is cross-entropy. However, the focus is not on the network's predictive capacity. We just use it to train the network parameters and so the final hidden layer's parameters serve as the learned static functional connectivity features, as shown in the static feature in Fig 1.

Fig 2. The entire process of processing static functional connectivity.

For dynamic FC, we can know that each object corresponds to a matrix of $116 \times 116 \times 40$ after PCA processing, as shown in Fig 3. The first step is channel compression. As mentioned before, each 116×116 layer corresponds to a distinct frequency. The compression of channels can be interpreted as the neural network looking for the most discriminative frequency bands for classification, which may have certain reference value for the analysis of fMRI data. Hence, in comparison to the extraction of features from static FC, the processing of dynamic FC necessitates an additional step of channel compression. The first layer comprises 1 filter with a corresponding 1×1 convolutional kernel. Then it is sent to the BN layer and the ReLu layer. The left operations is similar to the operations that performs on the static FC. Finally the dynamic features required in the Fig 1 are obtained.

Fig 3. The entire process of processing dynamic functional connectivity.

Subsequently, SRS features are concatenated with those gleaned from both static FC and dynamic FC. This composite feature set is then submitted to a SVM with a linear kernel for further classification. This experiment uses a 10-fold cross-validation of the data. The reason why this study uses SVMs instead of the multi-layer perceptron (MLP) for classification is because in experiments we find that the MLP model has poor generalization capability and performs poorly on the test set. In the support vector machine part, we use the more common linear kernel for classification, because the extracted feature dimension is small enough compared to the data set. In order to illustrate the advantages of SVM, we also conduct experiments using MLP and RF for classification. The architecture of the MLP consists of two hidden layers, featuring 64 and 16 neurons respectively. The number of neurons in the input layer is the dimension after concatenating three features, and the output layer is 2 neurons. As for random forest, 100 trees are selected for classification. These experiments adopt 10-fold cross-validation, and the results are averaged after several experiments.

Results

0.5 Classification comparison

According to the results of cross-validation, sensitivity (SEN), specificity (SPE) and accuracy (ACC) are used as evaluation indicators for classification results. Here, true

positives (TPs) are considered to be correctly diagnosed ASD patients, true negatives (TNs) are considered to be truly diagnosed TCs, false positives (FPs) and false negatives (FNs) are respectively incorrectly diagnosed ASD patients and TCs. SEN, SPE and ACC are calculated as follows,

$$\begin{aligned} ACC &= \frac{TP+TN}{TP+FP+TN+FN} \\ SEN &= \frac{TP}{FN+TP} \\ SPE &= \frac{TN}{FP+TN}. \end{aligned}$$

The ACC shows the model's ability to predict correctly, while SEN and SPE tell the model's ability to identify subjects and negative examples respectively. After several ten-fold cross-validation, our framework achieves 94.30($\pm$0.73)% ACC, 95.14($\pm$1.59)% SEN and 94.15($\pm$2.24)% SPE. Fig 4 illustrates the performance of three different classifiers.

Fig 4. The performance of different classifiers.

At the same time, our model performs better compared to previous papers, as shown in Fig 5.

Fig 5. This figure compares the performance of this paper with previous papers. Wang [22]; Huang [23]; Yin [24]; Jiang [25]; Bhandage [26] .

0.6 Analysis

Previous research has revealed differences in the complex relationships between TCs and subjects with ASD, and that these differences in FC exist in multiple brain regions. To identify which brain regions may have undergone alterations, we leverage the convolutional layer weights in our selected model. This allows us to ascertain which interactions between brain areas have more pronounced impact on classification, thereby suggesting that these areas have important differences between autistic and normal individuals. Examining the network weight of the first layer of the static FC part and the network weight of the second layer of the dynamic FC part, we find that the corresponding weight is a matrix of 32×116. By taking the absolute value of the weight of the channel and summing it, we can obtain a matrix of 1×116. we then select the brain areas corresponding to the top 10 features with the largest absolute values as the most discriminative brain areas, as shown in Table 2 and Fig 6.

Fig 6. This figure shows the most discriminating brain areas.

As shown in Table 2, both static FC and dynamic FC in the heschl and superior frontal gyrus areas are considered to be one of the most effective areas for classification. We also find that both have a certain proportion in the cerebellum region [27, 28]. Previous classification models or physiological studies also have some similar conclusions that differences exist in these brain areas [29–32]. At the same time, based on the weights coming from the first convolution layer of dynamic FC part, we can also get the smaller frequency bands in which there may be greater differences in the brain activity between subjects with ASD and TCs. After analyzing the weights, we find that the frequency bands between 0.04231Hz-0.04769Hz and 0.06385Hz-0.067436Hz contribute

Table 2. The discriminating brain areas of static FC and dynamic FC.

Order	Static FC	Dynamic FC
1	SupraMarginal_L	Cerebellum_Crus2_R
2	Cingulum_Post_L	Thalamus_R
3	Cerebelum_Crus1_R	Cerebellum_3_R
4	Heschl_R	Heschl_R
5	Occipital_Inf_R	Temporal_Mid_L
6	Cerebelum_7b_L	Frontal_Inf_Oper_R
7	Cerebelum_9_R	Precentral_R
8	Cerebelum_3_R	Vermis_6
9	Frontal_Sup_Orb_R	Frontal_Sup_Orb_R
10	Temporal_Inf_R	Angular_R

more to classification. Perhaps there are more differences in brain activity between subjects with ASD and TCs in these frequency bands, which requires further physiological experimental confirmation.

Discussion

In pursuit of delineating the physiological disparities between individuals with ASD and TCs, we deploy a synergistic approach that melds both static FC and dynamic FC, defining specialized convolutional kernels for feature extraction from FC matrices. This method facilitates the identification of critical brain regions that are particularly contributory to distinguishing between the two groups–some of which have been corroborated by previous studies. Moreover, we discern two brain regions, the heschl and superior frontal gyrus, that contribute substantially to both static FC and dynamic FC for classifications. Additionally, we pinpoint narrower frequency bands where dynamic FC differences may exist. To further augment the diagnostic capabilities for autism, we incorporate SRS, combining them with FC features to auxiliary diagnose ASD effectively. In this way, our model achieves better classification results compared to some previous papers.

For future research, the integration of more diverse data sources for classification could be explored–for instance, by amalgamating various brain templates or utilizing time-frequency analysis methods to garner additional FC information. Another way is merging the text information obtained from communicating with patients and performing natural language processing to assist in screening. The introduction of these information not only advances the prospects of AI in medical diagnostics but also minimizes the likelihood of false positives or identifying those who pretend to be patients. In addition, the proposed model holds potential for aiding the diagnosis of other psychological conditions, such as depression.

Conclusion

In summary, this paper proposes a hybrid CNN-SVM network that combines autism early screening tools with resting-state fMRI data, achieving better classification performance. The model not only identifies brain areas that exert significant influence on classification outcomes but also elucidates the frequency bands that impact classification. These findings offer invaluable clues to the etiological mechanisms and the determination of biological markers for autism, and can also help diagnose patients with autism.

Acknowledgments

This work was supported by the National Science Foundation of China under Grant numbers 11671354.

References

1. Zeidan J, Fombonne E, Scorah J, Ibrahim A, Durkin M, Saxena S, et al. Global prevalence of autism: A systematic review update. Autism Research. 2022;15(5):778–790. doi:10.1002/aur.2696.

2. Mier W, Mier D. Advantages in functional imaging of the brain. Frontiers in Human Neuroscience. 2015;9. doi:10.3389/fnhum.2015.00249.

3. Daliri mr, Behroozi M. Advantages and Disadvantages of Resting State Functional Connectivity Magnetic Resonance Imaging for Clinical Applications. OMICS Journal of Radiology. 2014;3. doi:10.4172/2167-7964.1000e123.

4. Hutchison RM, Womelsdorf T, Allen EA, Bandettini PA, Calhoun VD, Corbetta M, et al. Dynamic functional connectivity: Promise, issues, and interpretations. NeuroImage. 2013;80:360–378. doi:https://doi.org/10.1016/j.neuroimage.2013.05.079.

5. Menon SS, Krishnamurthy K. A comparison of static and dynamic functional connectivities for identifying subjects and biological sex using intrinsic individual brain connectivity. Scientific reports. 2019;9(1):5729.

6. Patil AU, Ghate S, Madathil D, Tzeng OJ, Huang HW, Huang CM. Static and dynamic functional connectivity supports the configuration of brain networks associated with creative cognition. Scientific reports. 2021;11(1):165.

7. Volkmar FR. Encyclopedia of autism spectrum disorders. Springer; 2021.

8. Lyall K, Rando J, Toroni B, Ezeh T, Constantino JN, Croen LA, et al. Examining shortened versions of the Social Responsiveness Scale for use in autism spectrum disorder prediction and as a quantitative trait measure: Results from a validation study of 3–5 year old children. JCPP advances. 2022;2(4):e12106.

9. Borges L, Otoni F, Lima THd, Schelini PW. Social Responsibility Scale (SRS-2): Validity Evidence Based on Internal Structure. Psicologia: Teoria e Pesquisa. 2023;39:e39nspe11.

10. Kovacs Balint Z, Raper J, Michopoulos V, Howell LH, Gunter C, Bachevalier J, et al. Validation of the Social Responsiveness Scale (SRS) to screen for atypical social behaviors in juvenile macaques. PLOS ONE. 2021;16(5):1–19. doi:10.1371/journal.pone.0235946.

11. Craddock C, Benhajali Y, Chu C, Chouinard F, Evans A, Jakab A, et al. The neuro bureau preprocessing initiative: open sharing of preprocessed neuroimaging data and derivatives. Frontiers in Neuroinformatics. 2013;7(27):5.

12. Biswal B, Zerrin Yetkin F, Haughton VM, Hyde JS. Functional connectivity in the motor cortex of resting human brain using echo-planar MRI. Magnetic resonance in medicine. 1995;34(4):537–541.

13. Yan CG, Wang XD, Zuo XN, Zang YF. DPABI: data processing & analysis for (resting-state) brain imaging. Neuroinformatics. 2016;14:339–351.

14. Huang W, Goldsberry L, Wymbs NF, Grafton ST, Bassett DS, Ribeiro A. Graph Frequency Analysis of Brain Signals. IEEE Journal of Selected Topics in Signal Processing. 2016;10(7):1189–1203. doi:10.1109/JSTSP.2016.2600859.

15. Park YH, Cha J, Bourakova V, Lee JM. Frequency specific contribution of intrinsic connectivity networks to the integration in brain networks. Scientific reports. 2019;9(1):4072.

16. Grinsted A, Moore JC, Jevrejeva S. Application of the cross wavelet transform and wavelet coherence to geophysical time series. Nonlinear processes in geophysics. 2004;11(5/6):561–566.

17. Torrence C, Compo GP. A practical guide to wavelet analysis. Bulletin of the American Meteorological society. 1998;79(1):61–78.

18. Daffertshofer A, Lamoth CJ, Meijer OG, Beek PJ. PCA in studying coordination and variability: a tutorial. Clinical biomechanics. 2004;19(4):415–428.

19. Simonyan K, Zisserman A. Very deep convolutional networks for large-scale image recognition. arXiv preprint arXiv:14091556. 2014;.

20. Howard A, Sandler M, Chu G, Chen LC, Chen B, Tan M, et al. Searching for mobilenetv3. In: Proceedings of the IEEE/CVF international conference on computer vision; 2019. p. 1314–1324.

21. Niu Z, Zhong G, Yu H. A review on the attention mechanism of deep learning. Neurocomputing. 2021;452:48–62.

22. Wang C, Xiao Z, Wang B, Wu J. Identification of Autism Based on SVM-RFE and Stacked Sparse Auto-Encoder. IEEE Access. 2019;7:118030–118036.

23. Huang ZA, Zhu Z, Yau CH, Tan KC. Identifying Autism Spectrum Disorder From Resting-State fMRI Using Deep Belief Network. IEEE Transactions on Neural Networks and Learning Systems. 2021;32(7):2847–2861. doi:10.1109/TNNLS.2020.3007943.

24. Yin W, Mostafa S, Wu Fx. Diagnosis of Autism Spectrum Disorder Based on Functional Brain Networks with Deep Learning. Journal of Computational Biology. 2021;28(2):146–165. doi:10.1089/cmb.2020.0252.

25. Jiang W, Liu S, Zhang H, Sun X, Wang SH, Zhao J, et al. CNNG: A Convolutional Neural Networks With Gated Recurrent Units for Autism Spectrum Disorder Classification. Frontiers in Aging Neuroscience. 2022;14. doi:10.3389/fnagi.2022.948704.

26. Bhandage V, K MR, Muppidi S, Maram B. Autism spectrum disorder classification using Adam war strategy optimization enabled deep belief network. Biomedical Signal Processing and Control. 2023;86:104914. doi:https://doi.org/10.1016/j.bspc.2023.104914.

27. Sydnor LM, Aldinger KA. Structure, function, and genetics of the cerebellum in autism. Journal of psychiatry and brain science. 2022;7.

28. Laidi C, Neu N, Watilliaux A, Martinez-Teruel A, Razafinimanana M, Boisgontier J, et al. Preserved navigation abilities and spatio-temporal memory in individuals with autism spectrum disorder. Autism Research. 2023;16(2):280–293.

29. Prigge MD, Bigler ED, Fletcher PT, Zielinski BA, Ravichandran C, Anderson J, et al. L ongitudinal H eschl's Gyrus Growth During Childhood and Adolescence in Typical Development and Autism. Autism Research. 2013;6(2):78–90.

30. Patriquin MA, DeRamus T, Libero LE, Laird A, Kana RK. Neuroanatomical and neurofunctional markers of social cognition in autism spectrum disorder. Human brain mapping. 2016;37(11):3957–3978.

31. Arutiunian V, Gomozova M, Minnigulova A, Davydova E, Pereverzeva D, Sorokin A, et al. Structural brain abnormalities and their association with language impairment in school-aged children with Autism Spectrum Disorder. Scientific Reports. 2023;13(1):1172.

32. Banks MI, Krause BM, Berger DG, Campbell DI, Boes AD, Bruss JE, et al. Functional geometry of auditory cortical resting state networks derived from intracranial electrophysiology. PLOS Biology. 2023;21(8):1–44. doi:10.1371/journal.pbio.3002239.

Click here to access/download;Figure;Fig1.tif

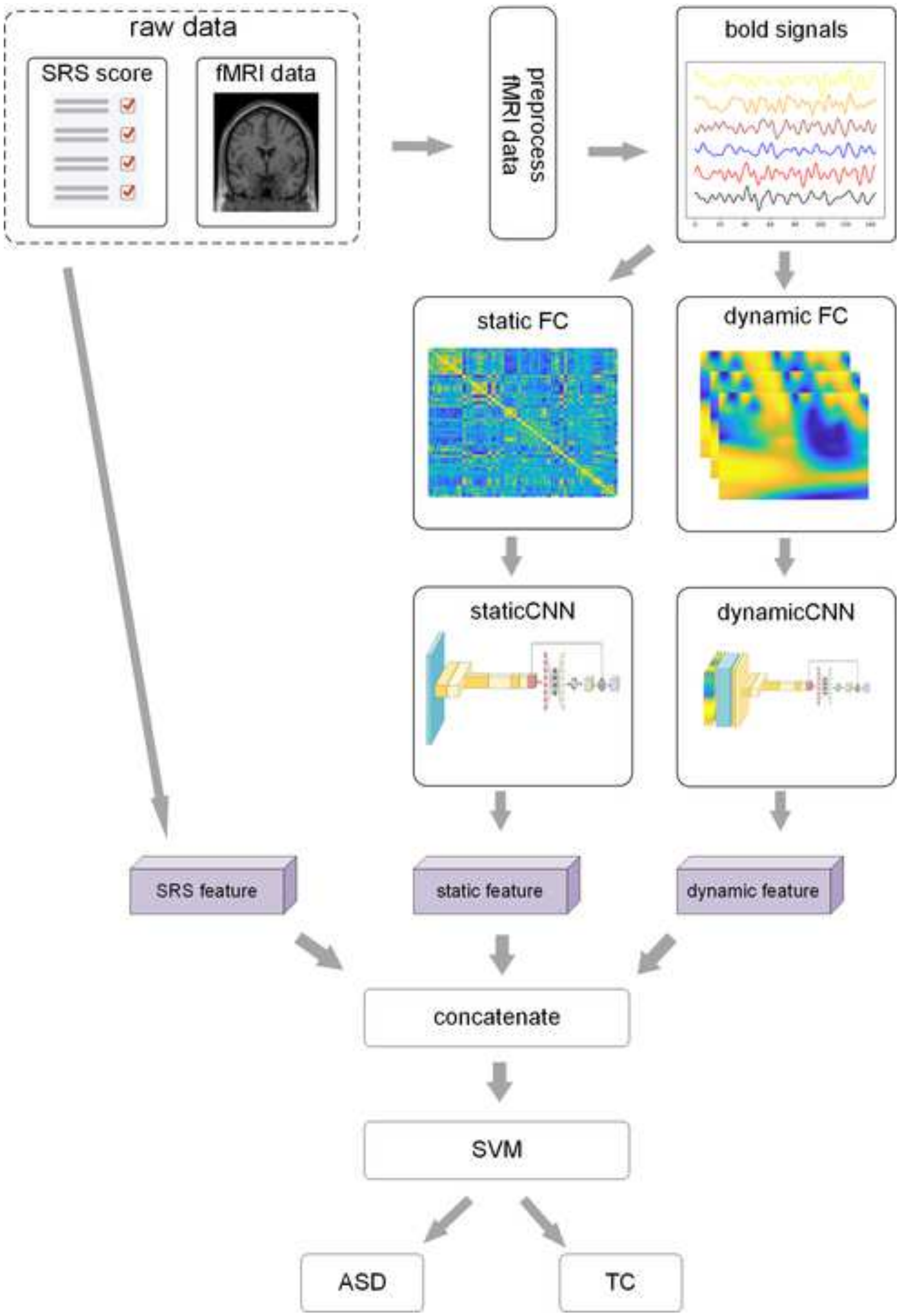

Click here to access/download;Figure;Fig2.tif

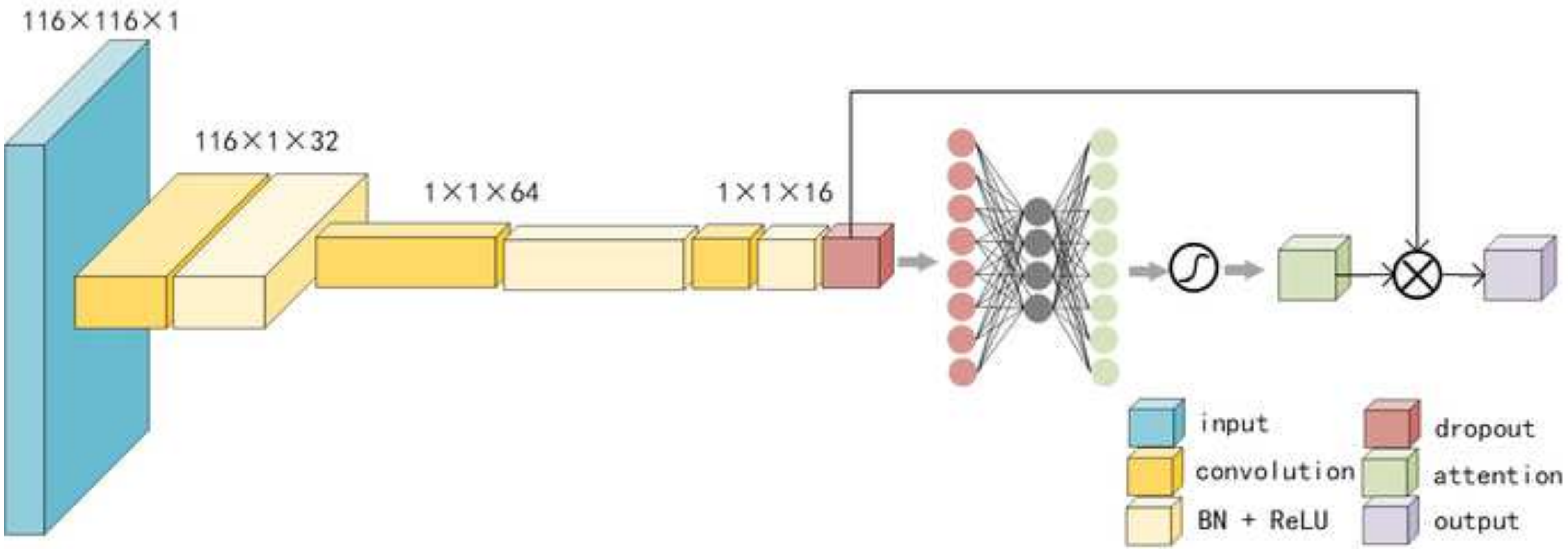

Click here to access/download;Figure;Fig3.tif

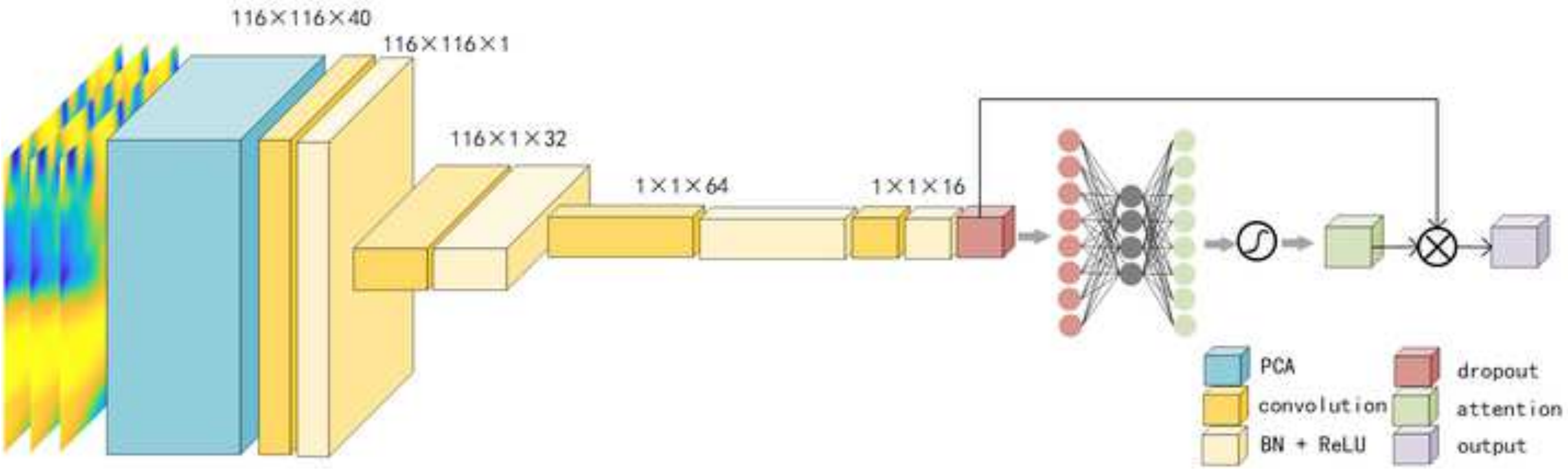

Click here to access/download;Figure;Fig4.tif

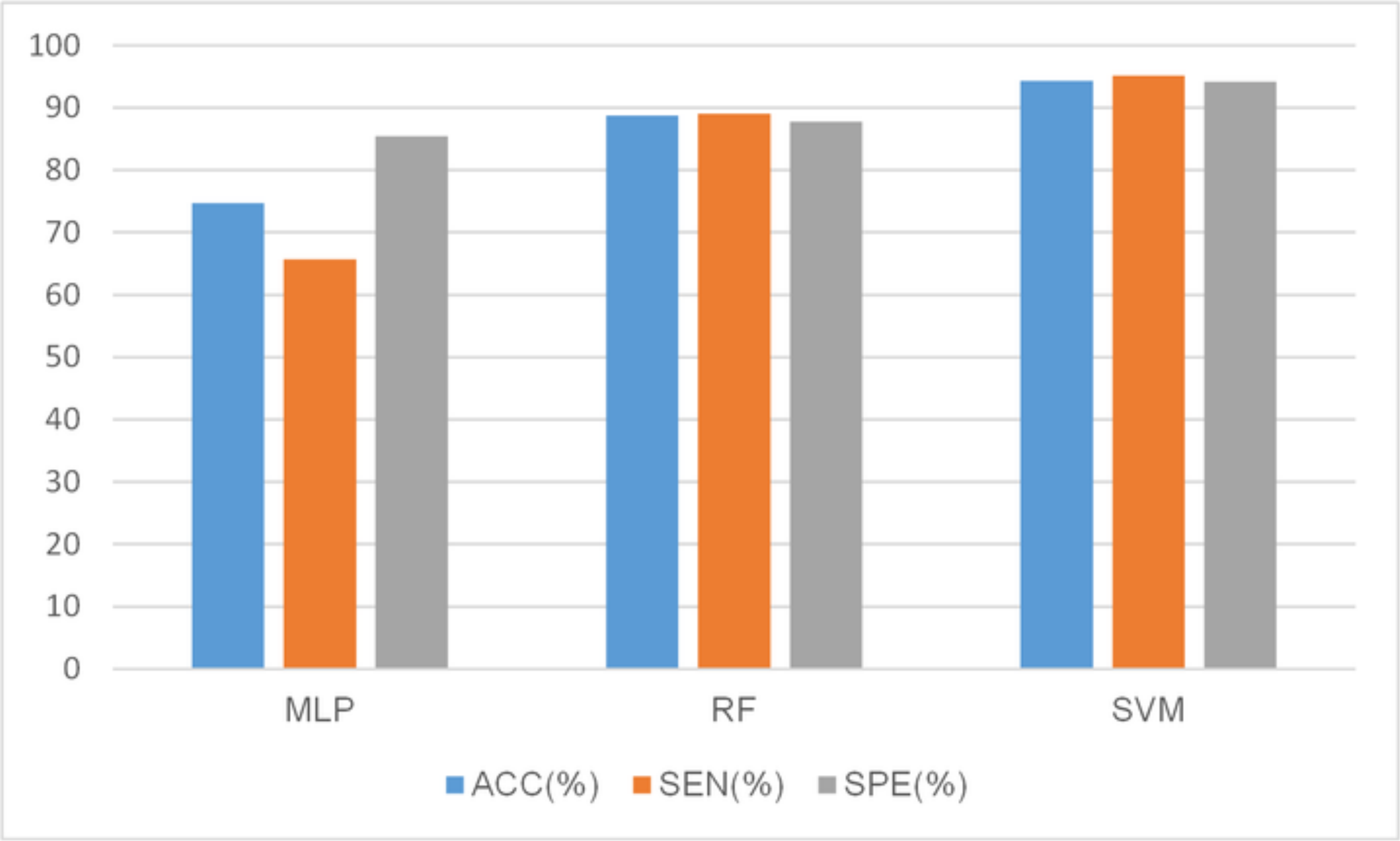

Click here to access/download;Figure;Fig5.tif

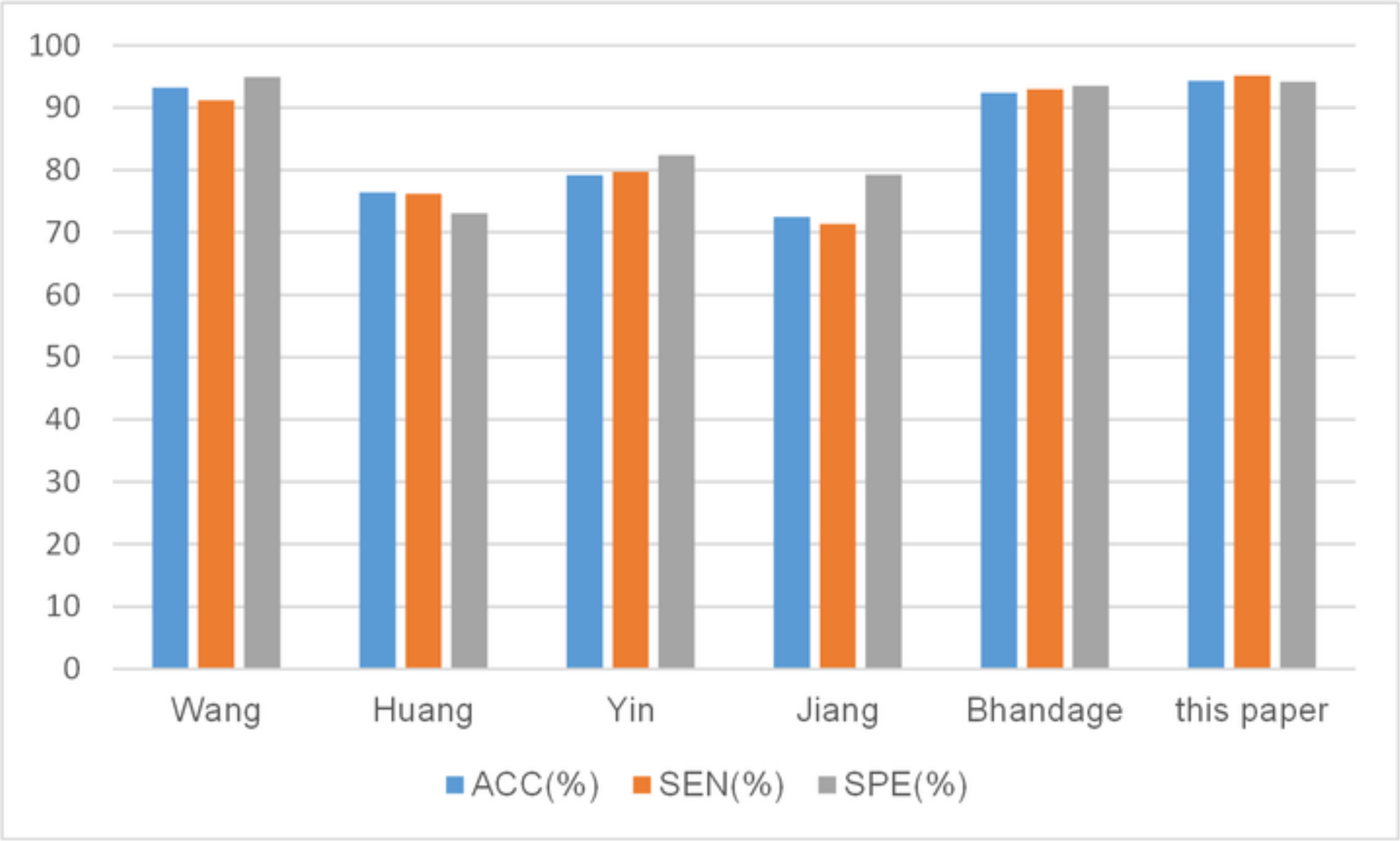

Click here to access/download;Figure;Fig6.tif

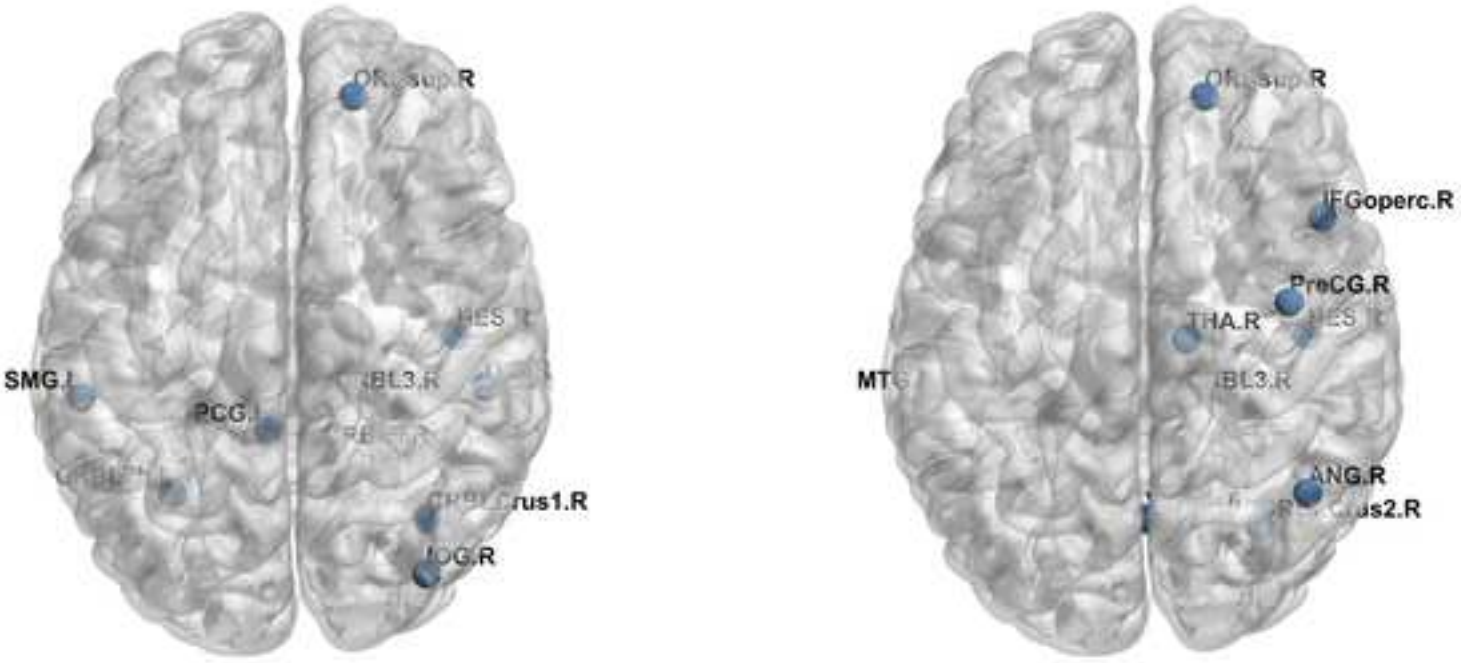

(a) (b)
