# Supplementary material for: A hybrid CNN-SVM model for enhanced autism diagnosis
Source: PLoS One. 2024 May 14;19(5):e0302236. doi: 10.1371/journal.pone.0302236 (PMC11093301; doi:10.1371/journal.pone.0302236)
Supplement: Attachment — Submitted filename: Response to Reviewers.pdf [file pone.0302236.s002.pdf]

Response Letter

Journal Title: PLOS ONE
Manuscript ID: PONE-D-23-32535
Title of Paper: A hybrid CNN-SVM model for enhanced autism diagnosis
Authors: Linjie Qiu, Jian Zhai

Dear Editor,

Thank you for your giving us an opportunity to revise our manuscript! Now we are submitting the revised manuscript entitled "A hybrid CNN-SVM model for enhanced autism diagnosis" for consideration for publication in PLOS ONE.

In the revision, we have performed nearly all the suggested opinions, experiments and analyses, and fully addressed the comments made by the reviewers and editor. The finished new experiments and data analyses as following:

1. We have revised the Abstract and Results sections to meet the requirements required for publication. And we add more related works in the Introduction section to review the application of machine learning in medical aspects, especially in the diagnosis of autism.

2. We have added the contributions of this work and the organization of this manuscript. We also have described the flowchart in algorithmic form to enhance the comprehension of our model.

3. In order to better evaluate the proposed model, we have introduced more indicators to evaluate the performance of different classifiers in autism diagnosis, as shown in the Fig 5.

4. We have analyzed the performance of the proposed model on different genders, sites, and age groups, and the results are shown in Figs 6-8.

5. We also used the Shapley value to measure the impact of three types of inputs on model predictions, giving interpretability to a certain extent.

6. We have added Limitation and Future prospects to the discussion section for better summary.

All authors have read and approved the re-submission of the manuscript! If you have any questions, please let me know!

Thank you for your consideration of our paper and we are looking forward to hearing from you!

Sincerely yours,

Linjie, Ph.D.,

School of Mathematical Sciences, Zhejiang University, Hangzhou, Zhejiang, China

Email:11935034@zju.edu.cn

1 Response to Editor

Comment-1. Please ensure that your manuscript meets PLOS ONE's style requirements, including those for file naming. The PLOS ONE style templates can be found at https://journals.plos.org/plosone/s/file?id=wjVg/PLOSOne_formatting_sample_main_body.pdf and https://journals.plos.org/plosone/s/file?id=ba62/PLOSOne_formatting_sample_title_authors_affiliations.pdf

RESPONSE: Thank you for your reminder. We have carefully reviewed our manuscript to ensure that it complies with PLOS ONE's style requirements, including file naming conventions. We used the LaTeX template provided by PLOS ONE, so there should be no issues with font and font size in the main body of the text. Furthermore, we have corrected any issues related to referencing figures and tables within the text to align with PLOS ONE's guidelines. Regarding the first page, we have made adjustments in accordance with the provided template, such as the citation matkers.

Comment-2. Please note that PLOS ONE has specific guidelines on code sharing for submissions in which author-generated code underpins the findings in the manuscript. In these cases, all author-generated code must be made available without restrictions upon publication of the work. Please review our guidelines at https://journals.plos.org/plosone/s/materials-and-software-sharing#loc-sharing-code and ensure that your code is shared in a way that follows best practice and facilitates reproducibility and reuse.

RESPONSE: We'd like to provide the code and the preprocessing information at github if this manuscript is accepted. We will make our code available without restrictions before publication.

Comment-3. Thank you for stating the following financial disclosure: "This work was supported by the National Natural Science Foundation of China under Grant numbers 11671354." Please state what role the funders took in the study. If the funders had no role, please state: "The funders had no role in study design, data collection and analysis, decision to publish, or preparation of the manuscript." If this statement is not correct you must amend it as needed. Please include this amended Role of Funder statement in your cover letter; we will change the online submission form on your behalf.

RESPONSE: We have added the statement "The funders had no role in study design, data collection and analysis, decision to publish, or preparation of the manuscript."

Comment-4. Thank you for stating the following in the Acknowledgments Section of your manuscript: “This work was supported by the National Science Foundation of China under Grant numbers 11671354.” We note that you have provided funding information that is not currently declared in your Funding Statement. However, funding information should not appear in the Acknowledgments section or other areas of your manuscript. We will only publish funding information present in the Funding Statement section of the online submission form. Please remove any funding-related text from the manuscript and let us know how you would like to update your Funding Statement. Currently, your Funding Statement reads as follows: “This work was supported by the National Natural Science Foundation of China under Grant numbers 11671354.” Please include your amended statements within your cover letter; we will change the online submission form on your behalf.

RESPONSE: We will provide funding information in the Funding Statement. And we have removed this funding-related text from the manuscript.

Comment-5. We note that Figures 1 and 6 in your submission contain copyrighted images. All PLOS content is published under the Creative Commons Attribution License (CC BY 4.0), which means that the manuscript, images, and Supporting Information files will be freely available online, and any third party is permitted to access, download, copy, distribute, and use these materials in any way, even commercially, with proper attribution. For more information, see our copyright guidelines: http://journals.plos.org/plosone/s/licenses-and-copyright.

RESPONSE: For Fig 1, we replace figures in SRS score part and fMRI data part. These figures comes from the website, https://pixabay.com/. And the figures we use meet the content license in https://pixabay.com/service/terms/. According to the CC0 License, the authors of that work have dedicated the work to the public domain by waiving all of his or her rights to the CC0 Content worldwide under copyright law, including all related and neighboring rights. Subject to the CC0 License Terms, the CC0 Content can be used for all personal and commercial purposes without attributing the author/ content owner of the CC0 Content or Pixabay. To meet publishing requirements, we add the sentences in the title of Fig 1 as follows, "The left figure in the raw data part is republished from [53] under a CC BY license, with permission from Pixabay, original copyright 2017. The right figure in the raw data part is republished from [54] under a CC BY license, with permission from Pixabay, original copyright 2015. " Other subfigures in Fig 1 are generated by ourself using matlab or visio.

For Fig 6, this figure is generated by matlab tool named Brainnet Viewer, which is a brain network visualization tool with the GNU General Public License. We overlooked citing this reference and have now added the citation in line 340 of the article.

2 Response to Reviewer #1

Comments: I think the authors need to do some efforts for comparing his approach with recent research studies such as in: https://onlinelibrary.wiley.com/doi/full/10.4218/etrij.2021-0097 You will find that this paper compares only one AutoML package from all available packages with Transfer Learning and Traditional Machine Learning. In addition, Linear Regression (LR) produce linear models which are similar to SVM and I am wondering why the authors did not compare the performance of the hybrid CNN-LR with CNN-SVM.

RESPONSE:For the former part of the suggestion, this article has indeed provided us with a perspective on classification based on facial image features, which we have incorporated into the introduction, in line 23 of the article. The sentence is "There are classification methods based on extracting features from facial image data[11], classification based on extracted features from audio and video data of subjects' behavior [12], classification based on subjects' eye-tracking data [13], and models based on functional magnetic resonance imaging (fMRI) data, etc."

Additionally, it has inspired us to consider incorporating such features for comprehensive classification, which we will treat as an assimilable feature in our future work, in line 403 of the article. The sentence is "Facial information of subjects can also be integrated for autism diagnosis [11]."

However, in conducting a literature comparison, the references we select for comparison are all based on the ABIDE dataset, whereas the article mentioned in the comments does not utilize the ABIDE dataset. We think that comparing with it may not be appropriate. To facilitate a more effective comparison between our proposed model and those presented in previous works, we have removed Fig 5 from the original manuscript. Instead, we have opted for the presentation of a table encompassing the method and feature details, which is Table 2 in the article.

For the latter part of the suggestion, we did not consider this at the time, so we have added a section about Linear Regression in Fig 5. And the description of LR is in line 289 of the article, that is, "And for LR, we choose a threshold of 0.5 to distinguish subjects with ASD and TCs."

3 Response to Reviewer #2

Comments: While the paper presents a novel and promising approach for diagnosing Autism Spectrum Disorder (ASD), it's important to acknowledge the limitations of the work. Some potential limitations of this research could include:

Comment-1. Keywords are missing in the abstract.

RESPONSE:Thank you for noticing this. We are using the latex template provided by PLOS ONE, and there is no keyword section in the template, so we did not use it. Also, keywords are not included in the given PLOS ONE template at

https://journals.plos.org/plosone/s/file?id=wjVg/PLOSOne_formatting_sample_main_body.pdf. Thus we think the keywords are not needed.

Comment-2. The sample size of 379 subjects with ASD and 442 typical controls, while substantial, may not be representative of the entire ASD population. The diversity of individuals with ASD in terms of age, gender, and severity of symptoms could impact the generalizability of the findings.

RESPONSE: We have chosen all the subjects with SRS scores from the ABIDE dataset. Currently we have no way to obtain more data, which may require more data to illustrate the performance of our model. In the Future prospects section, we mention that "This requires more experimental subjects to obtain a more generalizable result." in line 419.

We add a discussion on age and gender in the Classification comparison section in line 311. And the results are shown in Figs 6 and 7. The statement is as follows:

"Since the classification performance of the model can be affected by gender and age, we also analyze these two variables according to the results, as shown in Figs 6 and 7.

From Fig 6, we can see that the overall performance of women is better than men when the model makes predictions. The dataset used in this article involves 175 women and 646 men. This is because the prevalence rate in men is four times that of women, which may lead to an imbalance in the collected data. Thus, further confirmation of whether the model is better at predicting female subjects than men requires collecting more data. Overall, the performace of our model does not differ significantly across gender.

From Fig 7, we can see that the model's prediction results are very good in multiple age groups, and its performance is only slightly worse for subjects aged 20-30. This means that our model is less affected by age. "

However, the severity of symptoms is not reflected in the dataset, which is a limitation of our research and is mentioned in the Limitation section in line 393, which is as follows,

" Besides, autism has different severity levels, and these different levels of severity may exert an influence on categorization. Regrettably, our model does not reflect this well. Furthermore, our proposed model cannot infer the severity of the participants. In clinical practice, a comprehensive assessment, incorporating tools such as the diagnostic and statistical manual of mental disorders and clinical observations, is employed to definitively ascertain the severity of autism. Regrettably, our model does not have the ability to do this. "

Comment-3. The accuracy and reliability of the resting-state fMRI data can greatly influence the results. Issues such as motion artifacts, data preprocessing, and the consistency of data collection across different sites could affect the quality of the data.

RESPONSE: To address these concerns, we applied DPARSF for data preprocessing, aiming to minimize the potential problems introduced during this stage. We also ensured that there were no significant deviations observed in the data due to motion artifacts.

Regarding the variability introduced by data collection across different sites, this is an issue our model aims to tackle. To address this, we have figured the performance of our model based on data from each participating institution. As shown in Fig 8, our model performs well on datasets from different sites. And the description of this point is as follows in line 323 :

”At the same time, we also compare the prediction results of the model on data from different sites. As shown in Fig 8, we find that the model perform relatively well at most sites, but the performance is not very satisfactory at only two sites, ETH and IU. It shows that our model has a certain degree of robustness when processing data from different sites. ”

Comparing and classifying data from multiple sites may help account for additional sources of variation stemming from patient populations, scanning procedures and equipment differences, in contrast to a single-site dataset.

**Comment-4**. Deep learning models, including CNNs, have a risk of overfitting, especially when working with relatively small datasets. It's crucial to address how the model avoids overfitting and whether any techniques like cross-validation or regularization were used.

RESPONSE: We agree that addressing overfitting is crucial in our research, and we employed several strategies to mitigate this risk. These strategies included Batch Normalization, attention mechanisms, and cross-validation.

We integrate Batch Normalization into our model architecture to help stabilize and accelerate the training process. BN normalizes the input to each layer during training, which helps prevent internal covariate shift and reduces the likelihood of overfitting. This is implemented in line 242, 245 and 247 of the article.

Attention mechanisms are another key component of our model design, and they play a vital role in focusing on relevant information while suppressing noise in the data. This attention mechanism is applied at line 252 of the paper, and it contribute to the model's ability to generalize effectively.

To rigorously assess our model's performance and ensure its generalization across different data subsets, we employ ten-fold cross-validation techniques. Ten-fold cross-validation involves partitioning the dataset into multiple subsets and training and evaluating the model on different combinations of these subsets. This is discussed in line 101 and 290 of the article.

**Comment-5**. The model's performance might be optimized for the specific dataset and experimental setup used in the study. The paper should discuss the potential challenges in applying this model to new or different datasets.

RESPONSE: This perspective is valuable and we agree this is an important issue. Although our model performs well on datasets from different sites, when applied to unknown data, we may not be able to obtain the social information the model needs. When applied to other mental illnesses, we need new information for model training. This part is considered the limitation of our model, which is in line 388 of the discussion section in the manuscript. The statement is as follows,

” Meanwhile, our model also has certain limitations. When applying this model to a new dataset, it can be difficult to obtain social measures. When applying this model to other mental illnesses, such as depression, there are different perspectives and understandings when using related measures, which requires corresponding adjustments when applying the model. ”

Comment-6. The paper emphasizes the use of both static and dynamic functional connectivity features alongside SRS metrics. However, the paper should address whether the model's performance is heavily reliant on one type of feature and how generalizable these features are to other ASD populations.

RESPONSE: To solve the first problem, we introduce Shapley value. Shapley value was originally proposed in game theory to solve the problem of how to reasonably distribute benefits in multi-player cooperative games. In the field of machine learning, the Shapley value is used to explain the impact of each feature in the model on the prediction results. We add the absolute values of the Shapley values obtained for each feature corresponding to the three types of inputs to obtain their impact on model prediction. As can be seen from the Fig 10, the model does not rely too much on a certain type of features. A detailed introduction to shapeley value is in line 357 of the manuscript, which is as follows:

" Furthermore, in order to elucidate the contributions of SRS scores, static FC and dynamic FC to the model's predictions, we employ the concept of Shapley value. The Shapley value, derived from game theory, is utilized in allocating gains. In the realm of machine learning, Shapley values serve to expound the extent of influence each feature exerts on prediction outcomes within the model. For a given feature set S, the formula for calculating Shapley values is expressed as

$$\phi_S(x) = \sum_{T \subseteq S \setminus i} \frac{|T|!(|S| - |T| - 1)!}{|S|!}[f(x_T \bigcap i) - f(x_T)],$$

where i represents the feature to be calculated the Shapley value, T denotes the subset of S except i, x_T signifies the input containing only the features from T, and $x_T \bigcap i$ refers to the input containing features from both T and i. The meaning of this formula is that for any feature i, when it is added to a feature set S, its contribution to the final prediction result is calculated by combining i with other features in S.

In our specific case, for the purpose of comparing the contributions of the three types of inputs, we take the absolute values of the Shapley values computed for each subcategory of features and sum them. We have randomly selected several subjects, and the impact of SRS scores, static FC and dynamic FC on the output is illustrated in the Fig 10. As shown in Fig 10, the prediction of our model is not heavily dependent on one type of inputs. "

Regarding the generalizability of these features to other ASD populations, we acknowledge the importance of this aspect in the broader context of ASD research. The generalization ability of the model is reflected in its ability to adapt to fresh samples. In the 10-fold cross-validation experiment, we split the data into training and validation sets, and the results showed that our model performed well. This at least shows that the features learned by our model on the training set perform well on the validation set. However, whether it can be applied to other ASD population requires more data to verify, while this proecss may also occur other difficulties, which is discussed in line 411 of the Discussion section. The statement is as follows:

" Besides, although we use Shapley value as a visual display of model interpretability, there is no similar paradigm or formula that tells us why the model makes such a decision. It does not tell us how the model relates deep features to the inputs. The clinical interpretability of the model can be

figured out. In our research, we find that certain brain regions make a greater contribution to the diagnosis of autism. We also find that some previous articles also find differences in these regions. In clinical practice, further analysis may be possible, such as calculating the FC of these brain areas, and integrating the FC of multiple brain areas to obtain a threshold for distinguishing subjects with ASD from TCs. This requires more experimental subjects to obtain a more generalizable result. ”

Comment-7. While achieving a high classification accuracy is important, it's also crucial to consider the clinical interpretability of the model. Understanding the biological or clinical significance of the identified features and regions is essential for making practical use of the findings.

RESPONSE: In our research, we find that certain brain regions make a greater contribution to the diagnosis of autism. We also find that some previous articles also find differences in these regions. In the discussion section in line 411, we discuss that perhaps more precise detection of these regions can be done biologically or clinically, such as checking the functional connectivity of these regions and setting a threshold for differentiation. However, this requires further experiments for verification, and currently we are unable to conduct clinical experiments due to our lack of ability. The statement is as follows:

” Besides, although we use Shapley value as a visual display of model interpretability, there is no similar paradigm or formula that tells us why the model makes such a decision. It does not tell us how the model relates deep features to the inputs. The clinical interpretability of the model can be figured out. In our research, we find that certain brain regions make a greater contribution to the diagnosis of autism. We also find that some previous articles also find differences in these regions. In clinical practice, further analysis may be possible, such as calculating the FC of these brain areas, and integrating the FC of multiple brain areas to obtain a threshold for distinguishing subjects with ASD from TCs. This requires more experimental subjects to obtain a more generalizable result. ”

Comment-8. The paper should discuss the interpretability of the CNN-SVM model. Deep learning models are often seen as ”black boxes,” making it challenging to explain the model's decision-making process.

RESPONSE: In machine learning applications, the predictive power of a model is often superior, but this predictive power is often accompanied by a certain black-box nature, that is, it is difficult to understand why the model makes a specific decision. Understanding which input features have the greatest impact on a model's predictions is an important aspect of interpretability. In this article, we use Shapley value to describe the impact of three types of inputs on the model prediction results, which is described in Fig 10 and line 368 of the article. The output of the model is explained to some extent. The statement is as follows,

” In our specific case, for the purpose of comparing the contributions of the three types of inputs,

we take the absolute values of the Shapley values computed for each subcategory of features and sum them. We have randomly selected several subjects, and the impact of SRS scores, static FC and dynamic FC on the output is illustrated in the Fig 10. As shown in Fig 10, the prediction of our model is not heavily dependent on one type of inputs. ”

Of course, this is not enough to fully understand the decision-making process of the model. This aspect may be a future research direction, which is in line 411 as follows,

” Besides, although we use Shapley value as a visual display of model interpretability, there is no similar paradigm or formula that tells us why the model makes such a decision. It does not tell us how the model relates deep features to the inputs. The clinical interpretability of the model can be figured out. In our research, we find that certain brain regions make a greater contribution to the diagnosis of autism. We also find that some previous articles also find differences in these regions. In clinical practice, further analysis may be possible, such as calculating the FC of these brain areas, and integrating the FC of multiple brain areas to obtain a threshold for distinguishing subjects with ASD from TCs. This requires more experimental subjects to obtain a more generalizable result. ”

Comment-9. Any potential bias in the data collection, preprocessing, or in the model itself should be addressed. Additionally, the ethical considerations regarding the use of machine learning in healthcare, including issues related to privacy and consent, should be discussed.

RESPONSE: Potential Bias:First of all, in the data collection process, our selection criteria are objects that have both SRS score and fmri data, and all medical images have corresponding quality assessments (http://fcon_1000.projects.nitrc.org/indi/abide /ABIDEII_MRI_Quality_Metrics.zip). During data preprocessing, we did not find objects with particularly obvious offsets. In the process of data preprocessing, all operations are based on SPM12 and DPARSF. This part can be streamlined after the parameters are preset, and there should be no bias. Moreover, we acknowledge that model bias can be a concern, and we have taken steps to assess and mitigate it. This includes evaluating the model’s performance across different age ranges, sex and sites to identify and rectify any disparities, as shown in Figs 6, 7 and 8.

Ethical Considerations: First of all, there are corresponding instructions on the official website for the data we use, that is, in accordance with HIPAA guidelines and 1000 Functional Connectomes Project / INDI protocols, all datasets are anonymous, with no protected health information included (http://fcon_1000.projects.nitrc.org/indi/abide/abide_II.html). We can say that the dataset abides by ethical principles. We also do not violate the subjects’ privacy when we used the model to make predictions.

Comment-10. ASD is a developmental disorder, and it often involves changes in behavior and brain function over time. This study seems to focus on cross-sectional data. Longitudinal data might provide additional insights into how ASD evolves over time.

RESPONSE:Due to the limitations of our data set and the inability to collect the corresponding

data, we are unable to do this. We regard this research direction as part of future work, in line 421 of the article. The statement is as follows:

"Finally, since ASD is a developmental disorder characterized by potential fluctuations in behavior and brain function over time, longitudinal data could potentially offer additional insights into how ASD evolves over time. Subsequent scholars may consider seeking participants for months or even years of follow-up studies, meticulously documenting changes in their social behaviors and MRI data, thereby facilitating further study."

Comment-11. The study should discuss the validation methods employed, such as cross-validation, and the reproducibility of the results to ensure the robustness of the model's performance.

RESPONSE: We performed ten-fold cross-validation on the model in line 101, which is "The number of folds for cross-validation, denoted as $k = 10$." And in line 290, the statement is "These experiments all adopt the 10-fold cross-validation, and the results are averaged after several experiments. "

As for the reproducibility of the results, we provide the values of the hyperparameters in many places in the article to facilitate the reproduction of the model. At the same time, based on guidelines on code sharing for submissions of PLOS ONE, we will disclose the code on github after the article is accepted and make it available without restrictions to any reader before publication.

Comment-12. The authors used some evaluation metrics but they do not give us a clear understanding of the level of false positives and false negatives directly. Therefore, recently mean Intersection over union (mIoU) has been widely used as a more reasonable and intuitive way of metrics. mIoU should be highlighted in the Abstract and conclusion.

RESPONSE: mIoU (mean Intersection over Union) requires image segmentation tasks rather than classification tasks. mIoU is often used in pixel-level tasks such as image segmentation, where each pixel is assigned to a different class. In this case, IoU can be used to measure the segmentation performance of the model, and the IoU value of each category can be calculated, and then averaged to obtain mIoU. But for classification tasks, each sample is only assigned to one category and does not involve pixel-level segmentation, so mIoU is not an appropriate metric.

Here, we introduce the two indicators false negative rate (FNR) and false positive rate (FPR). FNR refers to the proportion of samples that the classification model incorrectly predicts as negative categories among the samples that are actually positive categories. FPR refers to the proportion of samples that the classification model incorrectly predicts as positive categories among the samples that are actually negative categories. At the same time, F1 score was also introduced to evaluate the performance of the classification model. It comprehensively considers the model's accuracy in predicting positive categories and its ability to identify positive category samples. The description are in line 294 as follows:

" According to the results of cross-validation, sensitivity (SEN), specificity (SPE), accuracy

(ACC), false positive rate (FPR), false negative rate (FNR) and F1 Score (F1) are used as evaluation indicators for classification results. Here, true positives (TPs) are considered to be correctly diagnosed ASD patients, true negatives (TNs) are considered to be truly diagnosed TCs, false positives (FPs) and false negatives (FNs) are respectively incorrectly diagnosed ASD patients and TCs. SEN, SPE and ACC are calculated as follows,

$$ACC = \frac{TP+TN}{TP+FP+TN+FN} \quad (1)$$

$$SEN = \frac{TP}{FN+TP} \quad (2)$$

$$SPE = \frac{TN}{FP+TN} \quad (3)$$

$$FPR = \frac{FP}{FP+TN} \quad (4)$$

$$FNR = \frac{FN}{FN+TP} \quad (5)$$

$$F1 = 2\frac{Precision * SEN}{Precision + SEN}, \quad (6)$$

where $Precison = TP/(TP + FP)$. Precision focuses on the model's accuracy in predicting positive examples. In order to balance the precision and SEN, the F1 score are used to comprehensively evaluate model performance. The ACC shows the model's ability to predict correctly, while SEN and SPE tell the model's ability to identify subjects and negative examples respectively. The FPR refers to the rate at which the model incorrectly classifies actual negative examples as positive examples. The FNR refers to the rate at which the model incorrectly classifies actual positive examples as negative examples. "

And the results are in Fig 5.

Comment-13. The proposed work must be compared with some state-of-the-art works from 2022/2023 for better understanding the capabilities of the proposed work.

RESPONSE: We add the comparison with some state-of-the-art works from 2022/2023 in Table 2. And we also add the information of the features and methods that those works use.

4 Response to Reviewer #3

Comment-1. Typing and grammatical errors are highlighted in the attached file

RESPONSE: Thank you for bringing the typing and grammatical errors to our attention. We appreciate your feedback, and we apologize for any oversights. We have fixed the typing and grammatical errors, which are not listed here. For statements that need to be reformulated, we modify them as follows:

1."However, when it comes to an individual FC matrix, each row or column corresponding to the correlation between one brain area and another brain area." → "However, when it comes to

the FC matrix for a subject, each row or column of the matrix corresponding to the correlation between two brain areas.” (line 231)

2.”The left operations is similar to the operations that performs on the static FC.” → The subsequent operations is similar to the operations that performed on the static FC. (line 275)

3.”As shown in Table 2, both static FC and dynamic FC in the heschl and superior frontal gyrus areas are considered to be one of the most effective areas for classification.” → ”As shown in Table 3, we list the top ten regions that contribute most to the classification in static FC and dynamic FC respectively. Among them, the heschl and superior frontal gyrus areas both play an important role in the classification based on two types of FC.” (line 344)

As for the highlighted parts in the Reference section, we are using the latex template provided by PLOS ONE and the BibTeX file provided by Google Scholar or the website where the article is published. We are not sure what the problem is about the citation of papers titled ”A comparison of static and dynamic functional connectivities for identifying subjects and biological sex using intrinsic individual brain connectivity” and ”Dynamic functional connectivity: Promise, issues, and interpretations ”. And we have corrected the page numbering error the paper titled ”Social Responsibility Scale (SRS-2): Validity Evidence Based on Internal Structure”

Comment-2. Unify all over the text Figure or Fig.

- 70 Figure 1.
- 175 Fig 2.
- 237 Fig 4 illustrates the performance of three different

RESPONSE: Thank you for pointing out the inconsistency in the usage of ”Figure” and ”Fig” throughout the text. We appreciate your attention to detail. According to PLOS ONE style template(https://journals.plos.org/plosone/s/file?id=wjVg/PLOSOne_formatting_sample_main_body.pdf), we use ”Fig” consistently throughout the manuscript for better readability and clarity.

Comment-3. Figure caption is not a sentence: • Fig. 5 This figure compares. . . .. A possible caption can be: ”Performance of the proposed model compared with related work (Wang [22]; Huang [23]; Yin [24]; Jiang [25]; and Bhandage [26])” • Fig 6. This figure shows the most discriminating brain areas. A possible caption can be: ”The most discriminating brain areas related to ASD” • Punctuation ruled should be respected.

RESPONSE: Thank you for your feedback regarding the figure captions. We agree that the figure captions should be improved for clarity and adherence to punctuation rules. We will make the necessary corrections to ensure that the figure captions are properly formatted and provide clear descriptions.

For Fig. 5, we have replaced it with a table containing the information of features and methods in previous papers and our model, as shown in Table 2.

For Fig. 6, the caption has been updated to: ”The most discriminating brain areas related to ASD.” And now it has become the title of Fig 9.

5 Response to Reviewer #4

Comments: Author presented a hybrid approach for autism spectrum disorder diagnosis. They made use of CNN and SVM based model to achieve this. But, there are several drawbacks in the article that should be resolved.

Comment-1. Abstract is not clear. It should express research backround , problem statement, methods and results.

RESPONSE: We agree that the abstract should effectively convey the research background, problem statement, methods, and results. Thus, we rewrite our abstract as follows:

"Autism is a representative disorder of pervasive developmental disorder. It exerts influence upon an individual's behavior and performance, potentially co-occurring with other mental illnesses. Consequently, an effective diagnostic approach proves to be invaluable in both therapeutic interventions and the timely provision of medical support. Currently, most scholars' research primarily relies on neuroimaging techniques for auxiliary diagnosis and does not take into account the distinctive features of autism's social impediments. In order to address this deficiency, this paper introduces a novel convolutional neural network-support vector machine model that integrates resting state functional magnetic resonance imaging data with the social responsiveness scale metrics for the diagnostic assessment of autism. We selected 821 subjects containing the social responsiveness scale measure from the publicly available Autism Brain Imaging Data Exchange dataset, including 379 subjects with autism spectrum disorder and 442 typical controls. After preprocessing of fMRI data, we compute the static and dynamic functional connectivity for each subject. Subsequently, convolutional neural networks and attention mechanisms are utilized to extracts their respective features. The extracted features, combined with the social responsiveness scale features, are then employed as novel inputs for the support vector machine to categorize autistic patients and typical controls. The proposed model identifies salient features within the static and dynamic functional links, offering a possible biological foundation for clinical diagnosis. By incorporating the behavioral assessments, the model achieves a remarkable classification accuracy of 94.30%, providing a more reliable support for auxiliary diagnosis. "

Comment-2. Introduction section failed to mention the challenges of existing system, major contribution and structure of the manuscript.

RESPONSE: We agree that the introduction should provide a more comprehensive context for our research. To address this, we will make the necessary revisions to include the following elements:

Challenges of Existing System:

1. "However, for mental disorders like autism, a singular data source may be insufficient. Therefore, a more rational approach involves the integration of multiple facets through an ensemble learning model."(line 18)

2. "However, these methods are inevitably affected by clinical training, tools, and cultural background, thereby interfering with clinicians' subjective observations."(line 62)

Major Contribution (line 77):

”The contributions of this work are summarized as:

1. A novel ensemble learning model is proposed, which integrates information from FC in fMRI data with the SRS reflecting the degree of social impairment in subjects.

2. Leveraging fMRI data, we construct both static and dynamic FC. Based on the CNN structure of extracted features, the brain areas and frequency bands that play a greater role in classification are found. And the attention mechanism is introduced to enhance the extracted features from FC.

3. To assess performance, we conduct comparative evaluations with various machine learning models, RF, logistic regression (LR), multilayer perceptron (MLP) and SVM. We also show the model's performance on data across genders, age groups and sites. Besides, we compare our model with some previous works.”

Structure of the Manuscript (line 90):

”The structure of this paper is as follows. Section 2 introduces data preprocessing and network architecture. Section 3 presents our results. Section 4 discusses the limitations of our study and the future work. Section 5 provides the conclusions.”

Comment-3. Literature review is poor. Several works should be studied such as Deep Learning for Autism Diagnosis and Facial Analysis in Children, Conditional Generative Adversarial Network Approach for Autism Prediction, Deep learning-based feature selection and prediction system for autism spectrum disorder using a hybrid meta-heuristics approach, Machine learning for autism spectrum disorder diagnosis using structural magnetic resonance imaging: Promising but challenging

RESPONSE: The article titled Deep Learning for Autism Diagnosis and Facial Analysis in Children has been retracted, and so we think it is inappropriate to cite this article.

The article titled Conditional Generative Adversarial Network Approach for Autism Prediction introduces a new model, so we compare our model with this in Table 2 in the Classification comparison section.

The article titled Deep learning-based feature selection and prediction system for autism spectrum disorder using a hybrid meta-heuristics approach is not available to our school library freely. And from the abstract, we think it can be cited as a reference for diagnosis of autism. The statement is as follows:

”Nowadays, machine learning is widely employed in auxiliary diagnoses across various medical conditions, such as brain tumors [2,3], autism [4,5], depression [6] and more.” (line 9)

”In the context of autism, numerous studies have proposed various models from different perspectives, offering diverse strategies for diagnosis. There are classification methods based on extracting features from facial image data[11], classification based on extracted features from audio and video data of subjects' behavior[12], classification based on subjects' eye-tracking data[13], and models based on functional magnetic resonance imaging (fMRI) data[5], etc.” (line 22)

The article titled Machine learning for autism spectrum disorder diagnosis using structural magnetic resonance imaging: Promising but challenging is a review article. It gives us some

opinion about diagnosis of autism and difficulties. We mentioned this in the introduction section as follows:

"However, these methods are inevitably affected by clinical training, tools, and cultural background, thereby interfering with clinicians' subjective observations[24,25]."(line 62)

Comment-4. Proposed model can be expressed as an algorithm. Architecture of hybrid model can be represented.

RESPONSE: We have added the model as an algorithm in line 96 as follows:

" To enhance the comprehension of Fig 1, we shall describe the flowchart in algorithmic form as follows:

Input:

- Training dataset $D = \{(x_1, y_1), (x_2, y_2), ..., (x_n, y_n)\}$, where x_i denotes features, and y_i represents the corresponding labels.
- The number of folds for cross-validation, denoted as $k = 10$.

Output: A well-trained model.

Algorithm Description:

1. Preprocess the fMRI data x_i from the training dataset D, following the steps outlined in the subsection 0.1. This yields a new dataset $D_1 = \{(a_1, t_1, y_1), (a_2, t_2, y_2), ..., (a_n, t_n, y_n)\}$, where a_i represents SRS features, t_i corresponds to the ROI time series, and y_i denotes labels.

2. Based on t_i, calculate the required static FC b_i and dynamic FC c_i, with detailed computational formulas provided in subsections 0.2 and 0.3. This results in a new dataset $D_2 = \{(a_1, b_1, c_1, y_1), (a_2, b_2, c_2, y_2), ..., (a_n, b_n, c_n, y_n)\}$.

3. Partition the training dataset D_2 into k non-overlapping subsets, typically employing a K-fold cross-validation approach. For each fold i, designate it as the validation set while using the remaining $k - 1$ folds as the training set.

4. Feed the static FC b_i and dynamic FC c_i of the training set into a CNN for deep feature extraction. Utilize the backpropagation algorithm and Adam optimizer to update model parameters. Employ the cross-entropy loss function, monitor its convergence during training, and set the maximum number of epochs to 50. The details of neural network architecture can be found in subsection 0.4. Assuming the extracted deep features are denoted as d_i and e_i, combine them with a_i as (a_i, d_i, e_i) and feed them into a linear kernel SVM for training.

5. Utilize the well-trained model to make predictions on the validation set and compute performance metrics.

6. Repeat steps 4 and 5 iteratively, using each fold as the validation set, to obtain a set of performance metrics.

7. Calculate the average performance metrics from the k folds to assess the model's performance. Concurrently, save the model parameters from the training process with the best performance metrics as the final model for deployment.

"

Comment-5. What is the need for devising hybrid model?. Mention the significance of incorporating SVM with CNN model.

RESPONSE: We hope to use different kinds of information of the same subject for integrated learning, so relying solely on the brain's MRI data is not enough. Thus, we choose to use CNN to extract deep features about static FC and dynamic FC, and combine them with other features for classification. To this end, the SRS score is introduced used for classification. After merging features, it is found that the effect of using multi-layer perceptron is not ideal. After trying several types of machine learning methods, svm is adopted. The performance of different classifiers is shown in Fig 5.

Comment-6. Several dataset can be used for experimentation. Hyper parameter tuning needs to be explained.

RESPONSE: Our dataset is fMRI data from different institutions. It is a synthesis of multiple small data sets. In the preprocessing stage of fMRI data, we use DPARSF, and the information related to its processing will be made public on GitHub. We can make public the parameters of the selected processing steps through matlab files, and the information of the corresponding machines of each institution through the word file (including the type of the machine and the time of sampling). , slice number, etc.). The hyperparameter adjustment for functional connection using CNN is manually selected and tried based on experience. The article mentions some hyper-parameter settings, including alpha values (line 251, $\alpha = 0.01$). Besides, based on guidelines on code sharing for submissions of PLOS ONE, we will disclose the code on github after the article is accepted and make it available without restrictions to any reader before publication.

Comment-7. Discussion section can be included to state the challenges, inferences and future prospects.

RESPONSE: We have add the Limitation and Future prospects in the Conclusion section as follows: ”

Discussion

In pursuit of delineating the physiological disparities between individuals with ASD and TCs, we deploy a synergistic approach that melds both static FC and dynamic FC, defining specialized convolutional kernels for feature extraction from FC matrices. This method facilitates the identification of critical brain regions that are particularly contributory to distinguishing between the two groups–some of which have been corroborated by previous studies. Moreover, we discern two brain regions, the heschl and superior frontal gyrus, that contribute substantially to both static FC and dynamic FC for classifications. Additionally, we pinpoint narrower frequency bands where dynamic FC differences may exist. To further augment the diagnostic capabilities for autism, we

incorporate SRS, combining them with FC features to auxiliary diagnose ASD effectively. In this way, our model achieves better classification results compared to some previous papers.

5.1 Limitation

Meanwhile, our model also has certain limitations. When applying this model to a new dataset, it can be difficult to obtain social measures. When applying this model to other mental illnesses, such as depression, there are different perspectives and understandings when using related measures, which requires corresponding adjustments when applying the model.

Besides, autism has different severity levels, and these different levels of severity may exert an influence on categorization. Regrettably, our model does not reflect this well. Furthermore, our proposed model cannot infer the severity of the participants. In clinical practice, a comprehensive assessment, incorporating tools such as the diagnostic and statistical manual of mental disorders and clinical observations, is employed to definitively ascertain the severity of autism. Regrettably, our model does not have the ability to do this.

5.2 Future prospects

For future research, the integration of more diverse data sources for classification could be explored–for instance, by amalgamating various brain templates or utilizing time-frequency analysis methods to obtain additional FC information. Facial information of subjects can also be integrated for autism diagnosis. Another way is merging the text information obtained from communicating with patients and performing natural language processing to assist in screening. The introduction of these information not only advances the prospects of AI in medical diagnostics but also minimizes the likelihood of false positives or identifying those who pretend to be patients. In addition, the proposed model holds potential for aiding the diagnosis of other psychological conditions, such as depression.

Besides, although we use Shapley value as a visual display of model interpretability, there is no similar paradigm or formula that tells us why the model makes such a decision. It does not tell us how the model relates deep features to the inputs. The clinical interpretability of the model can be figured out. In our research, we find that certain brain regions make a greater contribution to the diagnosis of autism. We also find that some previous articles also find differences in these regions. In clinical practice, further analysis may be possible, such as calculating the FC of these brain areas, and integrating the FC of multiple brain areas to obtain a threshold for distinguishing subjects with ASD from TCs. This requires more experimental subjects to obtain a more generalizable result.

Finally, since ASD is a developmental disorder characterized by potential fluctuations in behavior and brain function over time, longitudinal data could potentially offer additional insights into how ASD evolves over time. Subsequent scholars may consider seeking participants for months or even years of follow-up studies, meticulously documenting changes in their social behaviors and MRI data, thereby facilitating further study. ”

Comment-8. Conclusion section can be improved.

RESPONSE: We appreciate your suggestion for improvement, and we agree that the conclusion should be enhanced for better clarity and effectiveness. We have revised the conclusion, briefly reviewed our methods and findings, briefly summarized existing limitations and future prospects, and summarized the contributions of our article, as follows: " In summary, this article offers a valuable insights into autism prediction. The proposed model transcends the confines of a singular data type, presenting a hybrid CNN-SVM network model that combines ASD early screening tools with resting-state brain fMRI data. This model demonstrates good performance in classifying data across different genders, age groups and sites. Moreover, using FC, the model not only identifies brain regions with significant influence on classification outcomes but also elucidates the frequency bands that impact classification. These findings may offer clues to the etiological mechanisms and the determination of biological markers for autism. This research extends the comprehension of autism diagnosis models, encouraging comprehensive predictive analysis through the integration of multifaceted information.

Nevertheless, the article harbors limitations, such as its inability to provide predictions regarding the severity of autism and the difficulties of applying the model to new datasets. These shortcomings necessitate future research to overcome.

Future studies may delve into the integration of more information to characterize subjects or explore autism's temporal evolution from a longitudinal perspective. This endeavor promises to further enrich our understanding of autism and significantly enhance the diagnosis of autism.

In conclusion, this study's significance lies in the introduction of a hybrid CNN-SVM model that integrates social metrics and fMRI data. It charts a promising method for future research, which is integrating diverse sources of information for prediction. "

6 Response to Reviewer #5

Comments: The contributions is clear. However, authors should justify the decisions that made in the article such as the selected methods and parameters settings. The authors should state the parctical implications of the work.

RESPONSE: We appreciate your comment, and we recognize the importance of providing clear justifications for our decisions in the article. To address this, we make the following improvements:

In order to measure the performance of each classifier, we use multiple statistical indicators and compare the results in Fig 5, and conclude that using SVM for classification has better results. The related information is in line 294 as follows:

" According to the results of cross-validation, sensitivity (SEN), specificity (SPE), accuracy (ACC), false positive rate (FPR), false negative rate (FNR) and F1 Score (F1) are used as evaluation indicators for classification results. Here, true positives (TPs) are considered to be correctly diagnosed ASD patients, true negatives (TNs) are considered to be truly diagnosed TCs, false positives (FPs) and false negatives (FNs) are respectively incorrectly diagnosed ASD patients and

TCs. SEN, SPE and ACC are calculated as follows,

$$
\begin{aligned}
ACC &= \frac{TP+TN}{TP+FP+TN+FN} &(7)\\
SEN &= \frac{TP}{FN+TP} &(8)\\
SPE &= \frac{TN}{FP+TN} &(9)\\
FPR &= \frac{FP}{FP+TN} &(10)\\
FNR &= \frac{FN}{FN+TP} &(11)\\
F1 &= 2\frac{Precision * SEN}{Precision + SEN}, &(12)
\end{aligned}
$$

where $Precison = TP/(TP + FP)$. Precision focuses on the model's accuracy in predicting positive examples. In order to balance the precision and SEN, the F1 score are used to comprehensively evaluate model performance. The ACC shows the model's ability to predict correctly, while SEN and SPE tell the model's ability to identify subjects and negative examples respectively. The FPR refers to the rate at which the model incorrectly classifies actual negative examples as positive examples. The FNR refers to the rate at which the model incorrectly classifies actual positive examples as negative examples. "

We also use Shapley value to show that our model does not heavily depend on one kind of input, which also supports our method. The related information is in Fig 10 and in line 357 as follows:

"Furthermore, in order to elucidate the contributions of SRS scores, static FC and dynamic FC to the model's predictions, we employ the concept of Shapley value. The Shapley value, derived from game theory, is utilized in allocating gains. In the realm of machine learning, Shapley values serve to expound the extent of influence each feature exerts on prediction outcomes within the model. For a given feature set S, the formula for calculating Shapley values is expressed as

$$\phi_S(x) = \sum_{T \subseteq S \setminus i} \frac{|T|!(|S| - |T| - 1)!}{|S|!}[f(x_T \bigcap i) - f(x_T)],$$

where i represents the feature to be calculated the Shapley value, T denotes the subset of S except i, x_T signifies the input containing only the features from T, and $x_T \bigcap i$ refers to the input containing features from both T and i. The meaning of this formula is that for any feature i, when it is added to a feature set S, its contribution to the final prediction result is calculated by combining i with other features in S.

In our specific case, for the purpose of comparing the contributions of the three types of inputs, we take the absolute values of the Shapley values computed for each subcategory of features and sum them. We have randomly selected several subjects, and the impact of SRS scores, static FC and dynamic FC on the output is illustrated in the Fig 10. As shown in Fig 10, the prediction of our model is not heavily dependent on one type of inputs. "

As for parameters settings, provide the values of the hyperparameters in many places in the article to facilitate the reproduction of the model, such as "the number of folds for crossvalidation, denoted as k =10" (in line 101), "the dropout rate is set to 50%"(in line 250), "($alpha$ = 0.01"

(in line 251) and " with a learning rate set at 0.0001" (in line 262). These hyperparameters are manually adjusted, and the adjustment is based on the final statistics after 10-fold cross-validation. We think that the quality of experimental results can be used to justify the quality of parameter settings.

As for the parctical implications, We discuss this in the Future prospects subsection in line 411, as follows: "Besides, although we use Shapley value as a visual display of model interpretability, there is no similar paradigm or formula that tells us why the model makes such a decision. It does not tell us how the model relates deep features to the inputs. The clinical interpretability of the model can be figured out. In our research, we find that certain brain regions make a greater contribution to the diagnosis of autism. We also find that some previous articles also find differences in these regions. In clinical practice, further analysis may be possible, such as calculating the FC of these brain areas, and integrating the FC of multiple brain areas to obtain a threshold for distinguishing subjects with ASD from TCs. This requires more experimental subjects to obtain a more generalizable result."

7 Response to Reviewer #6

Comment-1: The abstract is adequate in length and structure.

RESPONSE: Thanks for your comment. To make the abstract better, we change it as follows:

"Autism is a representative disorder of pervasive developmental disorder. It exerts influence upon an individual's behavior and performance, potentially co-occurring with other mental illnesses. Consequently, an effective diagnostic approach proves to be invaluable in both therapeutic interventions and the timely provision of medical support. Currently, most scholars' research primarily relies on neuroimaging techniques for auxiliary diagnosis and does not take into account the distinctive features of autism's social impediments. In order to address this deficiency, this paper introduces a novel convolutional neural network-support vector machine model that integrates resting state functional magnetic resonance imaging data with the social responsiveness scale metrics for the diagnostic assessment of autism. We selected 821 subjects containing the social responsiveness scale measure from the publicly available Autism Brain Imaging Data Exchange dataset, including 379 subjects with autism spectrum disorder and 442 typical controls. After preprocessing of fMRI data, we compute the static and dynamic functional connectivity for each subject. Subsequently, convolutional neural networks and attention mechanisms are utilized to extracts their respective features. The extracted features, combined with the social responsiveness scale features, are then employed as novel inputs for the support vector machine to categorize autistic patients and typical controls. The proposed model identifies salient features within the static and dynamic functional links, offering a possible biological foundation for clinical diagnosis. By incorporating the behavioral assessments, the model achieves a remarkable classification accuracy of 94.30%, providing a more reliable support for auxiliary diagnosis. "

Comment-2. The indication of the dataset in the abstract must be ensured.

RESPONSE: The inclusion of dataset details in the abstract is essential. So we add the sentence ”We selected 821 subjects containing the social responsiveness scale measure from the publicly available Autism Brain Imaging Data Exchange dataset, including 379 subjects with autism spectrum disorder and 442 typical controls.”

Comment-3. Comparison with SOTA techniques should be mentioned in the abstract

RESPONSE: We appreciate the reviewer's suggestion to include a comparison with state-of-the-art (SOTA) techniques in the abstract. However, because we findd many different articles, these articles use different technologies, such as CNN and MLP, and claim that their results are sota. It may be tedious to mention this in the abstract. Therefore, we add Table 2 in the Classification comparison section to compare the features selected by different articles and the methods used.

Comment-4. The literature review of the study is insufficient, there are many important studies that are missing. The authors should include the following references with proper discussions:
https://doi.org/10.1109/iCareTech49914.2020.00032
https://doi.org/10.3390/app12083715
https://doi.org/10.3390/medicina58081090
https://doi.org/10.1038/s41598-023-30309-4
https://doi.org/10.1088/1742-6596/1916/1/012226
https://doi.org/10.1088/1757-899X/1055/1/012115
https://doi.org/10.1109/INDISCON50162.2020.00037
https://doi.org/10.1016/j.fcij.2017.12.001
https://doi.org/10.1016/j.cmpb.2019.05.015

RESPONSE: Thank you for your opinion. Since we basically only considered the use of the ABIDE data set during the study, we considered similar mental illnesses, but did not consider situations similar to brain tumors. We think that these articles can be used as examples of using machine learning and deep learning to deal with medical problems. Some of them can be introduced in the introduction as follows:

” Nowadays, machine learning is widely employed in auxiliary diagnoses across various medical conditions, such as brain tumors [2, 3], autism [4, 5], depression [6] and more. ” (line 9)

” These method include traditional machine learning techniques, such as support vector machine (SVM) [7], random forest (RF), and deep learning models, such as convolutional neural network (CNN) [8] and vision transformer (ViT) [9]. ” (line 11)

”When dealing with conditions characterized by lesions, such as brain tumors, a reliance on brain imaging data proves effective [10]. ” (line 17)

However, these articles cannot be used as examples for comparison in the classification comparison section. We have selected some other articles for comparison, as shown in table 2.

Comment-5. Please add contributions of your work in bulleted form preferably in the introduction section.

RESPONSE:Thank you for your valuable suggestion. We add the contributions of our work from line 77 as follows: "The contributions of this work are summarized as:

1. A novel ensemble learning model is proposed, which integrates information from FC in fMRI data with the SRS reflecting the degree of social impairment in subjects.

2. Leveraging fMRI data, we construct both static and dynamic FC. Based on the CNN structure of extracted features, the brain areas and frequency bands that play a greater role in classification are found. And the attention mechanism is introduced to enhance the extracted features from FC.

3. To assess performance, we conduct comparative evaluations with various machine learning models, RF, logistic regression (LR), multilayer perceptron (MLP) and SVM. We also show the model's performance on data across genders, age groups and sites. Besides, we compare our model with some previous works."

Comment-6. The organization of the article is missing.

RESPONSE: We add the contributions of our work from line 90 as follows: "The structure of this paper is as follows. Section 2 introduces data preprocessing and network architecture. Section 3 presents our results. Section 4 discusses the limitations of our study and the future work. Section 5 provides the conclusions."

Comment-7. There is irregular text in the article Lines: 62-63. Ensure a consistent heading number scheme. Correct it.

RESPONSE: Thank you for pointing out the inconsistency in the heading numbering scheme. According to the template given by PLOS ONE, we replace all the "Figure" with "Fig".

Comment-8. How you are claiming CNN static, rather the converse is true.
Comment-9. Defining static FC, you cannot change the working principle of CNN. Changing the name of the remarkable technique is no good. Please justify your approach.

RESPONSE: We appreciate your observation and would like to clarify that our claim relates to the utilization of static FC extracted by the convolutional neural network. And so we change the staticCNN and dynamic into CNN in Fig 1. Besides, the sentence "Details of the staticCNN and dynamicCNN part are illustrated in Figure 2 and Figure 3 separately" is changed into "Details of the CNN for extracting features from static and dynamic FC parts are illustrated in Figs 3 and 4 separately." in line 95-96.

**Comment-10**. Under the heading of Neural Network, do not use CNN. One is shallow and the other is deep.

RESPONSE:Thank you for your opinion. We change the section ”Neural Network” into ”CNN for feature extraction”.

**Comment-11**. Table 1 is without any whereabouts. The column headings, if possible, may be provided.

RESPONSE:Thank you for your opinion. We add the whereabouts and headings as shown in Table 1.

**Comment-12**. The distribution of instances in the dataset should be graphically represented.

RESPONSE: Thanks for your suggestion, we add a bar chart on the distribution of subjects as shown in Fig 2.

**Comment-13**. The figures related to CNN are not clearly represented according to the norms of deep learning. No figure number is there. Figures are contradictory and do not truly represent the working principle of algorithms.

RESPONSE: We appreciate your feedback regarding the figures related to CNN in our manuscript. After our inspection, we found that the number of filters in the third layer of the convolutional neural network was written incorrectly when the model extracted static FC. This may be because we mixed it with the effective neurons during training after the doupout layer when writing. Here we have modified it (in line 246). As for the figure number, since we are using a latex template, it will be automatically numbered when quoting the picture, so there should be no errors.

**Comment-14**. Bhandage has almost the same results as yours. Justify that the proposed solution would always be guaranteed through some statistical analysis.

RESPONSE: We appreciate the reviewer's comment regarding the similarity of our results to those of Bhandage. To ensure the robustness and guarantee the effectiveness of our proposed solution, we have performed ten-fold cross-validation, and the statement is as follows:

” The number of folds for cross-validation, denoted as k = 10. ” (line 101)

” These experiments all adopt the 10-fold cross-validation, and the results are averaged after several experiments. ” (line 290)

Besides, we choose more evaluation indicators, that is, sensitivity, specificity, accuracy, false positive rate, false negative rate and F1 Score. We use these metrics to evaluate the performance of our model across different age groups, genders and sites, as shown in Fig 6, Fig 7 and Fig 8, thus making a comprehensive evaluation of our model. The statement is as follows:

” According to the results of cross-validation, sensitivity (SEN), specificity (SPE), accuracy

(ACC), false positive rate (FPR), false negative rate (FNR) and F1 Score (F1) are used as evaluation indicators for classification results. Here, true positives (TPs) are considered to be correctly diagnosed ASD patients, true negatives (TNs) are considered to be truly diagnosed TCs, false positives (FPs) and false negatives (FNs) are respectively incorrectly diagnosed ASD patients and TCs. SEN, SPE and ACC are calculated as follows,

$$ACC = \frac{TP+TN}{TP+FP+TN+FN} \quad (13)$$

$$SEN = \frac{TP}{FN+TP} \quad (14)$$

$$SPE = \frac{TN}{FP+TN} \quad (15)$$

$$FPR = \frac{FP}{FP+TN} \quad (16)$$

$$FNR = \frac{FN}{FN+TP} \quad (17)$$

$$F1 = 2\frac{Precision * SEN}{Precision + SEN}, \quad (18)$$

where $Precison = TP/(TP+FP)$. Precision focuses on the model's accuracy in predicting positive examples. In order to balance the precision and SEN, the F1 score are used to comprehensively evaluate model performance. The ACC shows the model's ability to predict correctly, while SEN and SPE tell the model's ability to identify subjects and negative examples respectively. The FPR refers to the rate at which the model incorrectly classifies actual negative examples as positive examples. The FNR refers to the rate at which the model incorrectly classifies actual positive examples as negative examples. After ten-fold cross-validation, our framework achieves 94.30% ACC, 92.87% SEN, 95.47% SPE, 7.12%FPR, 4.52% FNR and 94.73% F1. Fig 5 illustrates the performance of different classifiers.

Since the classification performance of the model can be affected by gender and age, we also analyze these two variables according to the results, as shown in Figs 6 and 7

From Fig 6, we can see that the overall performance of women is better than men when the model makes predictions. The dataset used in this article involves 175 women and 646 men. This is because the prevalence rate in men is four times that of women, which may lead to an imbalance in the collected data. Thus, further confirmation of whether the model is better at predicting female subjects than men requires collecting more data.

From Fig 7, we can see that the model's prediction results are very good in multiple age groups, and its performance is only slightly worse for subjects aged 20-30. This means that our model is less affected by age.

At the same time, we also compare the prediction results of the model on data from different sites. As shown in Fig 8, we find that the model perform relatively well at most sites, but the performance is not very satisfactory at only two sites, ETH and IU. It shows that our model has a certain degree of robustness when processing data from different sites. ”

Comment-15. Limitations of the proposed work with future recommendations should be added before the conclusion.

RESPONSE: We agree that it is important to discuss the limitations of our proposed work and provide future recommendations. Thus, we add these two parts in the discussion section as follow:"

7.1 Limitation

Meanwhile, our model also has certain limitations. When applying this model to a new dataset, it can be difficult to obtain social measures. When applying this model to other mental illnesses, such as depression, there are different perspectives and understandings when using related measures, which requires corresponding adjustments when applying the model.

Besides, autism has different severity levels, and these different levels of severity may exert an influence on categorization. Regrettably, our model does not reflect this well. Furthermore, our proposed model cannot infer the severity of the participants. In clinical practice, a comprehensive assessment, incorporating tools such as the diagnostic and statistical manual of mental disorders and clinical observations, is employed to definitively ascertain the severity of autism. Regrettably, our model does not have the ability to do this.

7.2 Future prospects

For future research, the integration of more diverse data sources for classification could be explored–for instance, by amalgamating various brain templates or utilizing time-frequency analysis methods to obtain additional FC information. Facial information of subjects can also be integrated for autism diagnosis. Another way is merging the text information obtained from communicating with patients and performing natural language processing to assist in screening. The introduction of these information not only advances the prospects of AI in medical diagnostics but also minimizes the likelihood of false positives or identifying those who pretend to be patients. In addition, the proposed model holds potential for aiding the diagnosis of other psychological conditions, such as depression.

Besides, although we use Shapley value as a visual display of model interpretability, there is no similar paradigm or formula that tells us why the model makes such a decision. It does not tell us how the model relates deep features to the inputs. The clinical interpretability of the model can be figured out. In our research, we find that certain brain regions make a greater contribution to the diagnosis of autism. We also find that some previous articles also find differences in these regions. In clinical practice, further analysis may be possible, such as calculating the FC of these brain areas, and integrating the FC of multiple brain areas to obtain a threshold for distinguishing subjects with ASD from TCs. This requires more experimental subjects to obtain a more generalizable result.

Finally, since ASD is a developmental disorder characterized by potential fluctuations in behavior and brain function over time, longitudinal data could potentially offer additional insights into how ASD evolves over time. Subsequent scholars may consider seeking participants for months or even years of follow-up studies, meticulously documenting changes in their social behaviors and MRI data, thereby facilitating further study."

Comment-16. The Conclusion needs rephrasing, as it is not encircling the proposed work being asked for publication.

RESPONSE: We have rephrased and revised the Conclusion section to better describe the significance, limitations and contributions of our proposed work, and the future work, ensuring it aligns more closely with the focus of the publication request. The new version is as follows:

"In summary, this article offers a valuable insights into autism prediction. The proposed model transcends the confines of a singular data type, presenting a hybrid CNN-SVM network model that combines ASD early screening tools with resting-state brain fMRI data. This model demonstrates good performance in classifying data across different genders, age groups and sites. Moreover, using FC, the model not only identifies brain regions with significant influence on classification outcomes but also elucidates the frequency bands that impact classification. These findings may offer clues to the etiological mechanisms and the determination of biological markers for autism. This research extends the comprehension of autism diagnosis models, encouraging comprehensive predictive analysis through the integration of multifaceted information.

Nevertheless, the article harbors limitations, such as its inability to provide predictions regarding the severity of autism and the difficulties of applying the model to new datasets. These shortcomings necessitate future research to overcome.

Future studies may delve into the integration of more information to characterize subjects or explore autism's temporal evolution from a longitudinal perspective. This endeavor promises to further enrich our understanding of autism and significantly enhance the diagnosis of autism.

In conclusion, this study's significance lies in the introduction of a hybrid CNN-SVM model that integrates social metrics and fMRI data. It charts a promising method for future research, which is integrating diverse sources of information for prediction."
