# Supplementary material for: A hybrid CNN-SVM model for enhanced autism diagnosis
Source: PLoS One. 2024 May 14;19(5):e0302236. doi: 10.1371/journal.pone.0302236 (PMC11093301; doi:10.1371/journal.pone.0302236)
Supplement: Attachment — Submitted filename: Response to Reviewers.pdf [file pone.0302236.s003.pdf]

Response Letter

Journal Title: PLOS ONE
Manuscript ID: PONE-D-23-32535R1
Title of Paper: A hybrid CNN-SVM model for enhanced autism diagnosis
Authors: Linjie Qiu, Jian Zhai

Dear Editor,

Thank you for your giving us an opportunity to revise our manuscript! Now we are submitting the revised manuscript entitled "A hybrid CNN-SVM model for enhanced autism diagnosis" for consideration for publication in PLOS ONE.

In the revision, we have performed nearly all the suggested opinions and fully addressed the comments made by the reviewers and editor. We summarized the modifications as follows:

1. We have checked all references and can ensure that none of them have been withdrawn.

2. We have adjusted the images using the Preflight Analysis and Conversion Engine (PACE) digital diagnostic tool to ensure they meet PLOS requirements.

3. We have made appropriate revisions to the content based on the reviewers' comments.

All authors have read and approved the re-submission of the manuscript! If you have any questions, please let me know!

Thank you for your consideration of our paper and we are looking forward to hearing from you!

Sincerely yours,

Linjie, Ph.D.,
School of Mathematical Sciences, Zhejiang University, Hangzhou, Zhejiang, China
Email:11935034@zju.edu.cn

1 Response to Editor

Comment-1. Please review your reference list to ensure that it is complete and correct. If you have cited papers that have been retracted, please include the rationale for doing so in the manuscript text, or remove these references and replace them with relevant current references. Any changes to the reference list should be mentioned in the rebuttal letter that accompanies your revised manuscript. If you need to cite a retracted article, indicate the article's retracted status in the References list and also include a citation and full reference for the retraction notice.

RESPONSE: We have checked on the website where the references were published and can ensure that none of them have been withdrawn.

2 Response to Reviewer #6

Comments-1: In line number 80 (page 37), replace "...SRS scores, which reflect the..." with "....SRS scores reflecting the...".

RESPONSE: Thank you for your feedback. We noticed that there may be ambiguity in this sentence. The sentence " A novel ensemble learning model is proposed, which integrates information from FC in fMRI data and the SRS scores, which reflect the degree of social impairment in subjects." is changed into " A novel ensemble learning model is proposed, which integrates information from FC in fMRI data and the SRS scores. SRS scores reflect the degree of social impairment in subjects."

Comment-2. The features that we get from CNN are called dynamic features. In your work, this seems ambiguous, and one way to remove it is to change "static feature" in figure 1 (and throughout the article) to "staticFC features", and "dynamic feature" to "dynamicFC features"

RESPONSE: Thank you for your opinion. We change "static feature" into "staticFC features" in figure 1 and line 264. We change "dynamic feature" into "dynamicFC features" in figure 1 and line 275.

Comment-3. Remove the text lines in the conclusions "Nevertheless, the article harbors limitations, such as its inability...... diagnosis of autism." You have already mentioned this text in the limitations and future recommendations.

RESPONSE: Thank you for your opinion. We remove these text lines in the conclusions "Nevertheless, the article harbors limitations, such as its inability...... diagnosis of autism."
